# Extended Haplotypes in the Growth Hormone Releasing Hormone Receptor Gene (GHRHR) Are Associated with Normal Variation in Height

**DOI:** 10.1371/journal.pone.0004464

**Published:** 2009-02-11

**Authors:** Åsa Johansson, Inger Jonasson, Ulf Gyllensten

**Affiliations:** Department of Genetics and Pathology, Rudbeck laboratory, Uppsala University, Uppsala, Sweden; Vrije Universiteit Medical Centre, Netherlands

## Abstract

Mutations in the gene for growth hormone releasing hormone receptor (GHRHR) cause isolated growth hormone deficiency (IGHD) but this gene has not been found to affect normal variation in height. We performed a whole genome linkage analysis for height in a population from northern Sweden and identified a region on chromosome 7 with a lod-score of 4.7. The GHRHR gene is located in this region and typing of tagSNPs identified a haplotype that is associated with height (p = 0.00077) in the original study population. Analysis of a sample from an independent population from the most northern part of Sweden also showed an association with height (p = 0.0039) but with another haplotype in the GHRHR gene. Both haplotypes span the 3′ part of the GHRHR gene, including the region in which most of the mutations in IGHD have been located. The effect size of these haplotypes are larger than that of any gene previously associated with height, which indicates that GHRHR might be one of the most important genes so far identified affecting normal variation in human height.

## Introduction

Height is one of the most heritable traits in humans and since it is easy to measure has become one of the most studied phenotypes. Many genomic regions have been linked to the variation in stature, but few of these regions have been replicated [1]. SNPs in a number of genomic regions have recently been associated with human stature, but similar to the linkage analysis, few findings have been replicated between studies [2]–[4]. Although these associations have highly significant p-values, the effect size of each SNP is small and most of the loci contributing to variation in height still remain unidentified.

One approach to increase the power in linkage and association studies is to study small populations with increased linkage disequilibrium and decreased genetic diversity. In a population that has undergone a population bottleneck (or the founding of the population from a small number of individuals followed by rapid population growth) some alleles that were rare in the founding population have been maintained or even increased their frequency. One example is the Finnish population [5] were numerous rare diseases have been characterized, and for most of them a single gene and founding mutation has been identified [6]. Populations with this type of demographic history have been useful for identifying rare recessive alleles, but common variants with small effects have still been difficult to find. In an expanding population LD decays with time, while in a continuously small population LD is generated by genetic drift rather than founder effect. Small populations with a historic constant size may therefore be particularly suitable for identification of common disease alleles [7]. However, few human populations have had a demographic history that makes them candidates for such an analysis. One such population is the Sami population of the northern part of Scandinavia and Kola peninsula [8]. The Sami at present have an estimated population size of less than 100 000 individuals [9] and are believed to have populated the area soon after the last Glaciation, about 10 000 YBP. The LD in the Sami is increased relative to other many other populations [8], [10].

We have performed microsatellite marker based whole genome linkage analysis using a familial material from the southern Swedish Sami population. Linkage analysis was followed by typing of tagSNPs in the growth hormone releasing hormone receptor gene (GHRHR) located in the best linkage peak, both in the original population and in an independent northern Swedish population. A number of mutations in the GHRHR gene have previously been associated with isolated growth hormone deficiency (IGHD) leading to short stature, but no evidence has been found that GHRHR has an effect on normal variation in human stature.

## Results

### Linkage analysis of microsatellite markers

**Figure 1:**
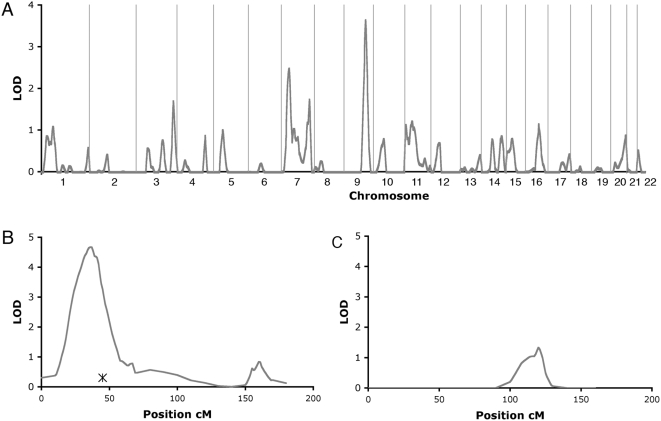
QTLs for height in the VB population. A) Multipoint LOD scores across the genome from the initial microsatellite scan. B) Multipoint LOD scores in the chromosome 7 and C) in the chromosome 9 region both including additional markers for fine-mapping. The location of the GHRHR gene is indicated by “X” in figure B.

The heritability for height in the VB cohort was estimated to 0.90 (p = 7.0E -23 Std. Error: 0.069), which is somewhat higher than in most populations [11]. In the genome-wide linkage analysis using 337 autosomal markers, two regions showed at least suggestive linkage to height (chromosome 7, lod = 2.42; and chromosome 9, lod = 3.58) (Figure 1). The peak on chromosome 7 was wide and many markers contributed to the linkage signal. Analysis of these and an additional 29 microsatellite markers (Supplementary material: Table S1) within 145 cM on chromosome 7 resulted in a highly significant lod score of 4.67 (Figure 1). The initial peak on chromosome 9 was due to three markers (d9s302, d9s930 and d9s938) and analysis of these and 12 additional markers within a 25 cM region reduced the lod from 3.58 to 1.33 (Figure 1).

### SNP association and haplotype scoring

We searched for candidate genes within the peak on chromosome 7 (Figure 1), starting at 11 cM and ending at 70 cM. This region contains as many as 394 genes. However we focused on the GHRHR gene which is of special interest since mutations in this gene cause isolated growth hormone deficiency (IGHD) [12]–[15]. In order to cover most of the genetic variation in the GHRHR gene, 9 tagSNPs within the gene and in the flanking regions were typed. Of the 9 SNPs, 4 were associated with variation in height (Table 1). Including all 9 markers, we identified one haplotype that was negatively associated with height, and that span the entire GHRHR gene region. This haplotype (characterized by the combination rs17159772-C, rs4988494-T, rs2267721-G, rs4988498-G, rs740336-C, rs2074780-G, rs4988504-G, rs4988505-G, rs2228078-T) had a frequency of 6.6% and could be perfectly tagged by 5 of the SNPs (rs17159772-C, rs4988494-T, rs2267721-G, rs4988498-G, rs4988505-G) (Table 2).

**Table 1:** TagSNPs in the GHRHR gene region and the estimated association to variation height in the VB and NB cohort.

Cohort	SNP name	Position	N	MAF	Beta*	P-value
NB	rs10265249	30952101	649	0.023	0.233	0.838
NB	rs4723034	30961436	651	0.242	0.235	0.437
VB	rs17159772	30969438	507	0.260	0.573	0.142
NB	rs17159772	30969438	696	0.242	−0.111	0.719
NB	rs2302019	30969948	651	0.306	0.177	0.514
VB	rs4988494	30970344	458	0.109	−1.160	0.093
NB	rs4988494	30970344	693	0.064	0.690	0.331
VB	rs2267721	30971968	497	0.328	−0.162	0.637
NB	rs2267721	30971968	695	0.257	−0.031	0.917
NB	rs2267723	30973467	651	0.487	0.334	0.057
VB	rs4988498	30976101	183	0.036	4.536	0.031
NB	rs4988498	30976101	651	0.004	6.727	0.023
VB	rs740336	30978202	506	0.020	0.123	0.938
VB	rs2074780	30980382	508	0.025	3.461	0.015
NB	rs2741	30982090	651	0.214	0.496	0.132
VB	rs4988504	30982543	506	0.065	0.829	0.317
VB	rs4988505	30983628	501	0.296	−0.372	0.011
NB	rs4988505	30983628	651	0.266	0.159	0.574
VB	rs2228078	30985377	506	0.026	−2.745	0.048

* Beta is the effect in cm for the minor allele.

**Table 2:** Association of the 5-marker haplotypes and height in the VB and NB cohort.

VB								
	rs17159772	rs4988494	rs2267721	rs4988498	rs4988505	Frequency	Hap-Score*	p-value
VB_hap#1	C	T	G	G	G	0.07	−3.36	0.00077
VB_hap#2	C	C	C	G	G	0.50	−1.43	0.15278
VB_hap#3	C	T	C	G	G	0.03	0.59	0.55240
VB_hap#4	T	C	G	G	C	0.14	0.82	0.41177
VB_hap#5	T	C	G	G	G	0.11	0.90	0.36837
VB_hap#6	C	T	C	G	C	0.02	1.32	0.18661
VB_hap#7	T	C	G	T	C	0.01	1.41	0.16001
VB_hap#8	C	C	C	G	C	0.10	1.88	0.06036
VB_hap#9	C	C	C	T	C	0.02	2.31	0.02088

NB								
	rs17159772	rs4988494	rs2267721	rs4988498	rs4988505	Frequency	Hap-Score*	p-value
NB_hap#1	T	C	G	G	G	0.09	−2.89	0.00391
NB_hap#2	C	C	C	G	G	0.59	−0.08	0.93779
NB_hap#3	C	T	G	G	G	0.01	0.03	0.97610
NB_hap#4	C	C	C	G	C	0.10	0.62	0.53658
NB_hap#5	T	C	G	G	C	0.16	0.78	0.43774
NB_hap#6	C	T	C	G	G	0.04	1.04	0.30049

* The haplotype-score is a relative measure of increase/decrease in average height for individuals carrying the haplotype.

### Replication attempt in an independent population

We used a population sample from the County of Norrbotten (NB), northern Sweden, which is part of the Northern Swedish Population Health Study, for an independent test of the association of the GHRHR haplotype with height. In total 10 SNPs were typed in NB, of which one (rs4988498) replicated the single SNP associations found in VB. However this SNP has a very low frequecy in NB and consequently the result could represent a false positive, even thought the effect is in the same direction in both cohorts. The 5 SNPs that tagged the haplotype negatively associated with height in VB also tagged a haplotype negatively associated with height in NB (Table 2). The two associated haplotypes in the VB and NB cohorts were found at similar frequency (6.6% vs. 8.7%) and were identical for the last three SNPs (rs2267721, rs4988498 and rs4988505).

**Figure 2:**
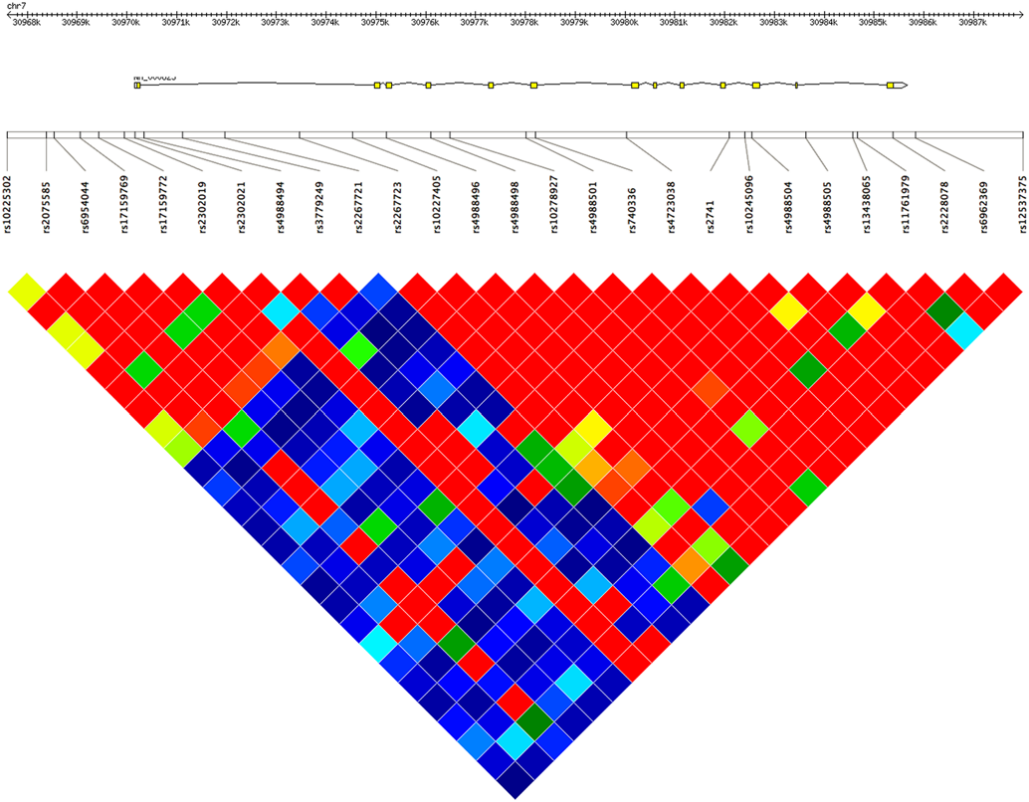
LD plot of the GHRHR gene region. Heat-map of the LD (r^2^) in the HapMap CEU population. The pairwise LD between SNPs and the location of the GHRHR gene.

The LD pattern in the GHRHR gene region in the HapMap CEU population clearly shows two blocks with high LD, separated by a region with low LD (Figure 2). The first block contains the first exon and most of the first intron, whereas the second LD block contains the remainder of the GHRHR gene. The two haplotypes associated with height in the VB and NB cohorts were identical for the two SNPs in the second LD block (rs4988498 and rs4988505) and for one SNP (rs2267721) located in between the blocks, but not for the SNPs within the first LD block (rs2302019 and rs2267723). This suggests that the SNPs affecting stature in the two cohorts are located downstream of the first intron of the GHRHR gene.

The p-values for the negatively associated 5-marker haplotypes were p = 7.6×10^−4^ and p = 3.9×10^−3^ in the VB and NB cohorts, respectively. For the VB cohort we also found one 5-marker haplotype that was positively associated with stature, but the frequency was low (2.4%) and no haplotype was positively associated in the NB cohort (Table 2).

### Haplotype diversity in a worldwide perspective

The haplotype diversity of the GHRHR gene region has previously been studied using the genome diversity panel, including 1,000 individuals from 52 populations, by analysis of 54 SNPs within a 53.4 kb region from about 28 kb upstream to 9 kb downstream of the gene (Supplementary material: Table S2). In the VB cohort the 5-marker haplotype that was negatively associated with height (Table 2) can be expanded to a 7-marker haplotype (rs17159772, rs4988494, rs2267721, rs4988498, rs740336, rs4988505 and rs2228078) where all SNPs overlapped with those typed in the diversity panel. The 7-marker haplotype negatively associated with height was found at a frequency of 6.6% in the VB cohort, and had a frequencies of at least 1% in the European diversity panel (Haplo_365). Similarly, the 5-marker haplotype negatively associated with height in the NB cohort (Table 2) can be expanded to a 10-marker haplotype (rs10278927, rs4723034, rs17159772, rs2302019, rs4988494, rs2267721, rs2267723, rs4988498, rs2741, rs4988505) that was present at a frequency of 4.8%. This haplotype matched two of the diversity panel haplotypes (denoted Haplo_28 and Haplo_388) occurring at frequencies of 4.4% and 1.9%, respectively, in the European populations.

The three haplotypes in the gene diversity panel (Haplo_365, 28 and 388) that matched the negatively associated haplotypes in the VB and NB cohorts were identical from markers rs4988494 to rs11760522, a region of more than 20 kb that includes 29 SNPs located throughout the entire GHRHR gene, from the second intron and downstream. A total of 12.7% of individuals of European descent in the gene diversity panel carry this extended haplotype.

We also identified one haplotype in the VB cohort that was positively associated with stature (p = 6.1×10^−3^) (Table 2). This haplotype matched Haplo_325 with a frequency of 3.2% in the gene diversity panel, which is similar to the frequency in VB (2.4%). As mentioned above the VB and NB haplotypes negatively associated with stature are identical for an extended haplotype. In contrast, European Haplo_325 in the diversity panel, which matched the VB haplotype positively associated with stature, differs at 11 out of the 29 positions.

### Haplotype effect

**Figure 3:**
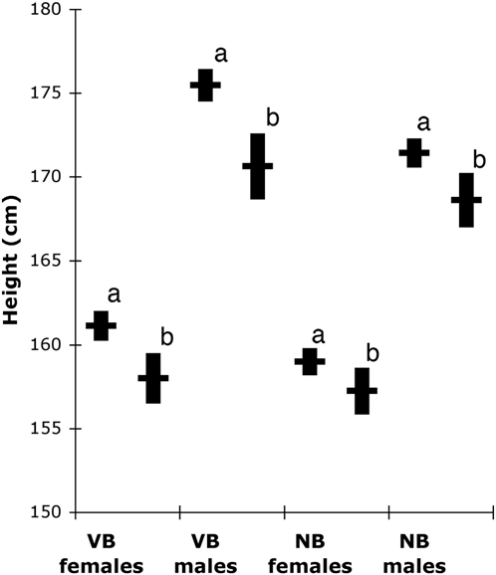
Effect of the haplotype on height. Average and standard error in height for individuals not carrying the haplotype associated with short stature (a) and individuals carrying the haplotype associated with short stature (b). The number of individuals in the group (a∶b) are in VB females 37∶230, VB males: 36∶218, NB females: 33∶338, NB males 30∶299.

We inferred haplotypes for each individual based on the 5-marker haplotypes (rs17159772, rs4988494, rs2267721, rs4988498 and rs4988505) and compared the height of individuals that are likely (probability>0.5) to carry the negatively associated haplotype to that of non-carriers (Figure 3). In the VB cohort the height of individuals carrying the associated haplotypes were 3.8 cm and 2.5 cm shorter for males and females, respectively, and in the NB cohort 2.1 cm and 1.2 cm shorter for males and females, respectively. After correcting for sex, age and population affinity, carrying or not carrying the negatively associated haplotype accounts for as much as 1.8% of the variation in height in our two populations (0.9% without adjustment).

## Discussion

We have identified a region on chromosome 7 linked to height in a family based study from northern Sweden. This chromosomal region has been proposed to be linked to height earlier [16], supporting our findings. The linkage region is large (about 60 cM) and gene-dense but includes one obvious candidate, the GHRHR gene, involved in growth hormone (GH) regulation. Since there are a total of 394 genes in the region, we cannot exclude that the linkage signal is due to variants in another gene. However, our analysis revealed an association between height and single markers as well as haplotypes in the GHRHR gene in the original Swedish population, as well an independent population sample. The two haplotypes negatively associated with height in the two Swedish populations are identical for three SNPs located in the first intron of the GHRHR gene and the downstream region. The 3′part of our two haplotypes, spanning from the first intron and downstream of the GHRHR gene, match a common haplotype that occurs at a frequency of 12.7% in European populations [17], [18].

Growth hormone (GH) is primarily involved in stimulating skeletal and visceral growth, but also has an important function in protein, lipid and carbohydrate metabolism. The synthesis and secretion of GH is mainly dependent on three hormones, GH-releasing hormone (GHRH), GH secretagogue (GHS) and somatostatin (SS). GHRH stimulates, via the GHRH receptor (GHRHR), GH synthesis and secretion. Isolated growth hormone deficiency (IGHD) has been clinically divided into three categories (types 1–3), although this subdivision does not take into account the molecular heterogeneity within each type. Patients with IGHD have presented with muations in either the GH, GHRHR or GH1 genes. A number of different mutations have been identified in the GHRHR gene leading to IGHD [12]–[15]. Mutations have been found in the promoter leading to reduced promoter activity [15] and the splice site of the first intron [13], resulting in changes of the concentration of components of the IGF (insulin-like growth factor) axis [19]. Five non-synonymous mutations have also been identified in familiar IGHD, at least one of which has been shown to result in loss of functionality of the GHRH receptor [12]–[15]. All 5 non-synonymous mutations are located within the boundaries of the extended haplotype region we found to be negatively associated with normal variation in height. Single SNPs and haplotypes in the GHRHR gene region have also been reported to be associated with normal short (not dwarfism) or tall stature, but these findings were not replicated in either of two independent population samples [20]. The haplotypes and all but one of the SNPs associated with height in the study by Lettre et al [20] are located upstream or in the promoter region of the GHRHR gene. The last SNP is located downstream of the gene, but their data does not include an analysis of haplotypes spanning the part of the GHRHR gene we found to be associated with height.

Linkage analysis is most suitable to detect rare variants with strong effects whereas association is most powerful to detect common variants with lower effects. TagSNPs chosen from the HapMap data are also optimized for detection of common variants [10]. To increase the power of tagSNPs to detect rare variants, more dynamic haplotype-based tagging methods can be used rather than single marker tests [21]. This agrees with our results showing that the single markers did not replicate the association of the GHRHR gene and normal variation in height and that the lowest p-values in the initial association analyses (p = 0.011) were not low enough to be remain significant after Bonferroni-correction for the 9 SNPs tested. Rather, analyses of 5-marker haplotypes was needed to detect a strong association with height. The similarity in frequency of the 5-marker haplotypes associated with height in the VB and NB cohorts, and the identity of the haplotype over a region of more than 20 kb, including 29 SNPs, suggests that if there is a causative variant that is shared between populations, it is relatively infrequent (<10%) and located downstream of the first intron.

The estimated effect size of the GHRHR haplotypes is considerably higher than that of other SNPs found to be associated with height [2]–[4], [22], [23]. For instance, the SNP in the HMGA2 gene region is estimated to explain 0.3% of the variation in height, and the average increase per risk allele is 0.4 cm. Our associated haplotype accounts for 1.8% of the variation in height after adjusting for sex, age and population affinity, and a decrease in height with as much as 1.2–3.8 cm, depending on sex and population cohort. The large effect size and the association of haplotypes in two independent populations, makes the GHRHR haplotype the strongest genetic contributor to normal variation human in height so far identified.

## Materials and Methods

### Samples

Samples for the linkage analysis (n = 436) were obtained from the southern Swedish Sami, County of Västerbotten (denoted the VB cohort). The individuals belong to 137 families. Association analysis was performed in the VB cohort and included 510 individuals, corresponding to the 436 individuals from the linkage analysis and an additional 74 individuals with no relatives present in the study. To replicate the association results, 699 samples were obtained from northern Sweden, County of Norrbotten (NB cohort) included in the Northern Swedish Population Health Study. A general description of the families, height, sex and age of the two populations can be found in Supplementary material: Table S3.

### Ethics Statement

This study was approved by the regional ethics committee.

### Marker selection

337 autosomal microsatellite markers (based on the Weber screening set 6: (http://research.marshfieldclinic.org/genetics/sets/Set6ScreenFrames.htm) were analyzed for the VB cohort. For fine mapping of two chromosomal regions, 29 additional markers on chromosome 7 and 12 markers on chromosome 9 (Supplementary material: Table S1) were selected from the NCBI (http://www.ncbi.nlm.nih.gov) and the ENSEMBL (http://www.ensembl.org) databases. SNP selection was based on genotype data from the CEU panel (Americans of European ancestry) of the phase II HapMap Project [24]. The SNPs were chosen to spatially cover and to capture most of the genetic variation at the candidate gene. For the SNP selection we used the pairwise tagging approach implemented in the Tagger software [21], including all SNPs with a frequency of more than 1% and using a threshold of 0.8 as the cut-off for two SNPs being tagged by each other. The 9 SNPs chosen (rs17159772, rs4988494, rs2267721, rs4988498, rs740336, rs2074780, rs4988504, rs4988505 and rs2228078) were estimated to capture 86% of all common variants (r^2^>0.8) within the GHRHR gene. For the replication, 5 SNPs tagging the haplotype in the VB cohort with the strongest association were chosen and an additional 5 SNPs (rs10265249, rs4723034, rs2302019, rs2267723 and rs2741) to further distinguish between different haplotypes in the diversity panel (Supplementary material: Table S2).

### Genotyping

#### Microsatellite markers

All markers were amplified using the same standard PCR protocol. For each 5 µl reaction, 10 ng DNA was used. The PCR contained 2.25 mM MgCl_2_, 200 µM of each dNTP, 0.2 µM of each primer and 0.125 u HotStarTaq DNA Polymerase. The thermocycle parameters used were: an initial 15 min denaturation at 95°C followed by 30 sec at 95°C, 30 sec at 55°C and 60 sec at 72°C for 35 cycles. For some markers from the additional two regions a 60-55°C touch down thermocycle program was used. All PCRs were performed using the ABI Prism™ 877 integrated thermal cycler. The forward primer of each marker was labeled with one of the following fluorescent dyes: HEX, NED or FAM. The labeled fragments were separated on an ABI Prism™ 3700 DNA Analyzer instrument. Genotype calling was performed using the AC genotyping software [25], the ABI GenoTyper™ or GeneMapper™ genotyping software.

#### SNP markers

SNPs were genotyped using TaqMan genotyping assays (Applied Biosystems) employing Applied Biosystems (AB) designed reagents mixes and oligonucleotide/primer pools, and the 7900 HT Fast Real Time PCR system (Applied Biosystems) according to the manufacturer's instructions.

### Statistic analysis

During quality control, all markers were checked for mendelian errors using the PedCheck software (http://watson.hgen.pitt.edu/register/docs/pedcheck.html) and markers that were not in Hardy-Weinberg equilibrium (p<0.05) were removed from further analyses. In all analyses, height trait was corrected for sex and age and transformed to be normally distributed with a mean of zero and standard deviation of one. Heritability was estimated using SOLAR software [26]

#### Linkage analysis

Linkage analysis was performed using SOLAR software [26]. Multipoint identical by descent matrixes were calculated every 1 cM along the chromosomes. Multipoint lod-scores were calculated in the first step every 10 cM throughout the genome and in the second step every 1 cM within all region with a lod-score above 0.56.

#### Association and haplotype scoring

Association analysis was performed using the GenABEL software [27]. For single markers, a score test was used for association between a trait and genetic polymorphism in samples of related individuals [28] based on the kinship matrix calculated based on the pedigree information. For haplotypes, maximum likelihood estimates of the haplotype frequencies and the posterior probabilities of the pairs of haplotypes for each subject were calculated using an EM algorithm implemented in the R-package haplo.stats version 1.3.1, followed by a score test for the association of haplotypes with the traits [29]. The fraction of the variance in height explained by the haplotypes were estimated by analysis of variance using a generalized linear model including sex, age, population affinity (VB or NB) and haplotype status (carrying or not carrying the negatively associated allele).

#### Haplotype diversity in a worldwide perspective

Haplotype frequencies for the Human Genome Diversity Panel [17], [18] were downloaded from the Fondation Jean Dausset - CEPH *World Wide Web* server (http://www.cephb.fr/) using the HGDP - CEPH Project - Genotype Database V2.0. The Human Genome Diversity Panel represents 52 populations with range of different histories.

## Supporting Information

Table S1 Microsatellite markers typed in the fine-mapping.
(0.05 MB DOC)

Table S2 The genotype and SNP infomration for the diversity panel can be found at the Fondation Jean Dausset - CEPH World Wide Web server (http://www.cephb.fr/). SNPs are colored as: Yellow: The overlap between VB, NB and diversity panel. Turquoise: The ovelap between VB and diversity panel. Pink: The overlap between NB and diversity panel. The haplotypes are colored as: Pink: The NB haplotype negatively associated with stature. Turquoise: The VB haplotype negatively associated with stature. Lilac: The VB haplotype positively associated with stature. Green: The most common haplotype both in NB (27%)and the Eurpean diversitypanel populations.
(0.16 MB PDF)

Table S3 General description of the two population cohorts
(0.05 MB DOC)
