# Supplementary material for: Extended Haplotypes in the Growth Hormone Releasing Hormone Receptor Gene (GHRHR) Are Associated with Normal Variation in Height
Source: PLoS One. 2009 Feb 11;4(2):e4464. doi: 10.1371/journal.pone.0004464 (PMC2637425; doi:10.1371/journal.pone.0004464)
Supplement: Table S1 — Microsatellite markers typed in the fine-mapping. (0.05 MB DOC) [file pone.0004464.s001.doc]

Table S1. Microsatellite markers typed in the fine-mapping.

| Marker name | Position (cM) |
| --- | --- |
| d7s488 | 29.28 |
| d7s2493 | 39.02 |
| d7s2416 | 41.69 |
| d7s2515 | 43.84 |
| d7s2496 | 47.08 |
| d7s484 | 53.5 |
| d7s528 | 57.79 |
| d7s691 | 63.67 |
| gck | 67.43 |
| d7s2427 | 68.5 |
| d7s670 | 69.56 |
| d7s674 | 70.64 |
| d7s2422 | 72.78 |
| d7s2429 | 76.71 |
| d7s672 | 84.52 |
| d7s1870 | 86.12 |
| d7s1797 | 93.63 |
| d7s804 | 94 |
| d7s2540 | 97.38 |
| d7s524 | 97.38 |
| d7s644 | 97.89 |
| d7s657 | 104.86 |
| d7s2459 | 119.81 |
| d7s2511 | 156.33 |
| d7s2426 | 160.09 |
| d7s642 | 162.33 |
| d7s3070 | 163.03 |
| d7s798 | 168.98 |
| d7s637 | 173.03 |
| d9s261 | 117.37 |
| d9s106 | 120.04 |
| d9s1856 | 120.04 |
| d9s1776 | 123.33 |
| d9s170 | 124.75 |
| d9s1872 | 129.74 |
| d9s258 | 130.52 |
| d9s1682 | 132.09 |
| d9s1881 | 135.85 |
| d9s1825 | 136.47 |
| d9s1821 | 137.35 |
| d9s1795 | 142.51 |
