# Supplementary material for: Extended Haplotypes in the Growth Hormone Releasing Hormone Receptor Gene (GHRHR) Are Associated with Normal Variation in Height
Source: PLoS One. 2009 Feb 11;4(2):e4464. doi: 10.1371/journal.pone.0004464 (PMC2637425; doi:10.1371/journal.pone.0004464)
Supplement: Table S2 — The genotype and SNP infomration for the diversity panel can be found at the Fondation Jean Dausset - CEPH World Wide Web server (http://www.cephb.fr/). SNPs are colored as: Yellow: The overlap between VB, NB and diversity panel. Turquoise: The ovelap between VB and diversity panel. Pink: The overlap between NB and diversity panel. The haplotypes are colored as: Pink: The NB haplotype negatively associated with stature. Turquoise: The VB haplotype negatively associated with stature. Lilac: The VB haplotype positively associated with stature. Green: The most common haplotype both in NB (27%)and the Eurpean diversitypanel populations. (0.16 MB PDF) [file pone.0004464.s002.pdf]

Haplotype nr	America	East Asia	Europe	Middle East	Central South Asia	Oceania	Subsaharian Africa	rs10265249	rs7458593	rs4723034	rs7384927	rs10225302	rs13438031	rs28371550	rs6954044	rs33995922	rs28371551	rs17159769	rs28371552	rs28371553	rs17159772	rs28371554	rs28371555	rs28371556	rs28371557	rs2302019	rs2302020	rs2302021	rs4988494	rs2267721	rs2267723	rs4988496	rs28371558	rs28371559	rs4988498	rs4988499	rs740336	rs28371560	rs2741	rs4988505	rs28371561	rs28371562	rs2228078	rs2586	rs28371563	rs28371564	rs28371565	rs28371566	rs6962369	rs10653361	rs4723039	rs28371567	rs28371568	rs28371569	rs28371570	rs11771444	rs11973385	rs11760522
Haplo_302	2.34%	31.47%	17.09%	9.51%	9.90%	14.29%	0%	1	1	1	1	1	1	1	1	1	1	1	1	1	1	1	1	1	1	1	1	1	1	1	1	1	1	1	1	1	1	1	1	1	1	1	1	1	1	1	1	1	1	2	1	1	1	1	1	1	1	2
Haplo_42	0%	1.08%	9.18%	4.29%	3.22%	30.36%	0.95%	1	1	1	2	2	2	2	1	1	1	2	2	1	2	1	1	1	1	2	2	1	1	2	2	1	1	1	1	1	1	1	1	2	1	1	1	1	1	1	1	1	1	1	1	2	1	1	2	1	1	2
Haplo_28	0%	0.22%	4.43%	3.99%	2.48%	1.79%	0%	1	1	1	2	2	2	2	1	1	1	2	2	1	2	1	1	1	1	2	2	1	1	2	1	1	1	1	1	1	1	1	1	1	1	1	1	1	1	1	1	1	1	2	1	1	1	1	1	1	1	2
Haplo_6	0.78%	0.43%	4.11%	1.53%	0.99%	0%	0%	1	1	1	2	1	1	1	1	1	1	1	1	1	1	1	1	1	1	1	1	1	1	1	1	1	1	1	1	1	1	1	1	1	1	1	1	1	1	1	1	1	1	2	1	1	1	1	1	1	1	2
Haplo_382	0%	0%	3.48%	6.44%	0.99%	0%	0%	1	1	1	1	2	1	1	1	2	1	1	1	1	1	1	1	1	1	2	1	1	2	1	2	1	1	1	1	1	1	1	2	1	1	1	1	1	1	1	1	1	2	1	1	1	1	1	1	2	2	1
Haplo_316	0%	0.22%	3.16%	1.23%	1.98%	1.79%	0%	1	1	1	1	1	1	1	1	1	1	1	1	1	1	1	1	1	1	1	1	1	1	1	2	1	1	1	1	1	1	1	1	2	1	1	1	1	1	1	1	1	1	1	1	2	1	1	2	1	1	2
Haplo_321	0.78%	4.31%	3.16%	1.53%	3.71%	0%	0%	1	1	1	1	1	1	1	1	1	1	1	1	1	1	1	1	1	1	1	1	1	1	1	2	1	1	1	1	1	1	1	2	1	1	1	1	1	1	1	1	1	2	1	1	1	1	1	1	2	2	1
Haplo_325	0%	0%	3.16%	3.07%	0.74%	0%	0%	1	1	1	1	1	1	1	1	1	1	1	1	1	1	1	1	1	1	1	1	1	1	1	2	2	1	2	2	1	1	1	1	2	1	1	1	1	1	1	1	1	1	1	1	2	2	1	1	2	2	1
Haplo_281	0%	0%	2.53%	2.15%	0.25%	0%	0%	2	2	2	2	1	1	1	2	1	1	1	1	1	1	1	1	1	1	1	1	1	1	1	1	1	1	1	1	1	1	1	1	1	1	1	1	1	1	1	1	1	1	2	1	1	1	1	1	1	1	2
Haplo_212	13.28%	1.08%	2.22%	0.31%	2.72%	7.14%	0%	1	2	2	2	1	1	1	1	1	1	1	1	1	1	1	1	1	1	1	1	1	1	1	1	1	1	1	1	1	1	1	1	1	1	1	1	1	1	1	1	1	1	2	1	1	1	1	1	1	1	2
Haplo_241	1.56%	0%	2.22%	0.31%	0.50%	0%	0%	1	2	2	2	2	2	2	1	1	1	2	2	1	2	1	1	1	1	2	2	1	1	2	1	1	1	1	1	1	1	1	1	1	1	1	1	1	1	1	1	1	1	2	1	1	1	1	1	1	1	2
Haplo_278	0%	0%	1.90%	0%	0%	0%	0%	2	2	2	2	1	1	1	1	1	1	1	1	1	1	1	1	1	1	1	1	1	1	1	2	1	1	1	1	1	1	1	1	2	1	1	1	1	1	1	1	1	1	1	1	2	1	1	2	1	1	2
Haplo_314	0%	0.43%	1.90%	0.31%	0.25%	0%	0%	1	1	1	1	1	1	1	1	1	1	1	1	1	1	1	1	1	1	1	1	1	1	1	2	1	1	1	1	1	1	1	1	1	1	1	1	1	1	1	1	1	1	2	1	1	1	1	1	1	1	2
Haplo_388	0%	1.08%	1.90%	2.76%	3.96%	1.79%	0%	1	1	1	1	2	2	2	1	1	1	2	2	1	2	1	1	1	1	2	2	1	1	2	1	1	1	1	1	1	1	1	1	1	1	1	1	1	1	1	1	1	1	2	1	1	1	1	1	1	1	2
Haplo_395	0%	0.65%	1.90%	1.53%	0%	3.57%	0%	1	1	1	1	2	2	2	1	1	1	2	2	1	2	1	1	1	1	2	2	1	1	2	2	1	1	1	1	1	1	1	1	2	1	1	1	1	1	1	1	1	1	1	1	2	1	1	2	1	1	2
Haplo_245	0%	0%	1.58%	1.23%	0%	0%	0%	1	2	2	2	2	2	2	1	1	1	2	2	1	2	1	1	1	1	2	2	1	1	2	2	1	1	1	1	1	1	1	1	2	1	1	1	1	1	1	1	1	1	1	1	2	1	1	2	1	1	2
Haplo_303	0%	0.22%	1.58%	0%	0%	0%	0%	1	1	1	1	1	1	1	1	1	1	1	1	1	1	1	1	1	1	1	1	1	1	1	1	1	1	1	1	1	1	1	1	1	1	1	1	1	1	1	1	1	1	2	1	1	1	1	1	2	2	1
Haplo_365	0%	2.59%	1.58%	1.53%	0.74%	0%	0%	1	1	1	1	2	1	1	1	1	1	1	2	1	1	1	1	1	1	2	1	2	2	2	1	1	1	1	1	1	1	1	1	1	1	1	1	1	1	1	1	1	1	2	1	1	1	1	1	1	1	2
Haplo_375	0%	1.29%	1.58%	2.76%	5.20%	0%	0%	1	1	1	1	2	1	1	1	2	1	1	1	1	1	1	1	1	1	2	1	1	2	1	1	1	1	1	1	1	1	1	1	1	1	1	1	1	1	1	1	1	1	2	1	1	1	1	1	1	1	2
Haplo_397	0%	0.43%	1.58%	0.92%	0.74%	0%	0%	1	1	1	1	2	2	2	1	1	1	2	2	1	2	1	1	1	1	2	2	1	1	2	2	1	1	1	1	1	1	1	2	1	1	1	1	1	1	1	1	1	2	1	1	1	1	1	1	2	2	1
Haplo_32	0%	0%	1.27%	0%	0%	0%	0%	1	1	1	2	2	2	2	1	1	1	2	2	1	2	1	1	1	1	2	2	1	1	2	1	1	1	1	1	1	1	1	1	2	1	1	1	1	1	1	1	1	1	1	1	2	1	1	2	1	1	2
Haplo_379	0%	0%	1.27%	2.45%	0.25%	0%	0%	1	1	1	1	2	1	1	1	2	1	1	1	1	1	1	1	1	1	2	1	1	2	1	2	1	1	1	1	1	1	1	1	1	1	1	1	2	1	2	1	1	2	1	1	2	1	1	1	2	2	1
Haplo_398	0%	0%	1.27%	0%	1.24%	0%	0%	1	1	1	1	2	2	2	1	1	1	2	2	1	2	1	1	1	1	2	2	1	1	2	2	1	1	1	1	2	2	1	1	2	1	1	1	1	2	1	1	1	1	1	1	2	1	1	1	2	2	1
Haplo_403	0%	0%	1.27%	1.23%	1.49%	0%	0%	1	1	1	1	2	2	2	1	1	1	2	2	1	2	1	1	2	1	2	2	1	1	2	2	1	1	1	1	1	1	1	1	2	1	1	1	1	1	1	1	1	1	1	1	2	1	1	2	1	1	2
Haplo_67	0%	0%	1.27%	0%	0.74%	0%	0%	1	1	1	1	1	1	1	1	1	1	1	1	1	1	1	1	1	1	1	1	1	1	1	1	1	1	1	1	1	1	1	1	1	1	1	1	1	1	1	1	1	1	2	1	1	1	1	1	1	1	2
Haplo_13	0%	0%	0.95%	1.23%	0%	0%	0%	1	1	1	2	1	1	1	2	1	1	1	1	1	1	1	1	1	1	1	1	1	1	1	1	1	1	1	1	1	1	1	1	1	1	1	1	1	1	1	1	1	1	2	1	1	1	1	1	1	1	2
Haplo_14	0%	0%	0.95%	0%	0%	0%	0%	1	1	1	2	1	1	1	2	1	1	1	1	1	1	1	1	1	1	1	1	1	1	1	2	1	1	1	1	1	1	1	1	1	1	1	1	1	1	1	1	1	1	2	1	1	1	1	1	1	1	2
Haplo_306	12.50%	0.22%	0.95%	0.31%	0.25%	0%	0%	1	1	1	1	1	1	1	1	1	1	1	1	1	1	1	1	1	1	1	1	1	1	1	1	1	1	1	1	1	1	1	1	1	1	1	2	1	1	1	1	1	1	2	2	1	1	1	1	1	1	2
Haplo_72	0%	0%	0.95%	0%	0.25%	0%	0%	1	1	1	1	1	1	1	1	1	1	1	1	1	1	1	1	1	1	1	1	1	1	1	2	2	1	2	2	1	1	1	1	2	1	1	1	1	2	1	1	1	1	1	1	2	1	1	1	2	2	1
Haplo_12	0%	0%	0.63%	0%	0.25%	0%	0%	1	1	1	2	1	1	1	1	1	1	1	1	1	1	1	1	1	1	1	1	1	1	1	2	1	1	1	1	1	1	1	2	1	1	1	1	1	1	1	1	1	2	1	1	1	1	1	1	2	2	1
Haplo_15	0%	0%	0.63%	0%	0%	0%	0%	1	1	1	2	2	1	1	1	1	1	1	1	1	1	1	1	1	1	1	1	1	1	1	1	1	1	1	1	1	1	1	1	1	1	1	1	1	1	1	1	1	1	2	1	1	1	1	1	1	1	2
Haplo_230	0%	0%	0.63%	0.31%	1.73%	0%	0%	1	2	2	2	2	1	1	1	2	1	1	1	1	1	1	1	1	1	2	1	1	2	1	1	1	1	1	1	1	1	1	1	1	1	1	1	1	1	1	1	1	1	2	1	1	1	1	1	1	1	2
Haplo_236	0%	0%	0.63%	0.31%	0.74%	0%	0%	1	2	2	2	2	1	1	1	2	1	1	1	1	1	1	1	1	1	2	1	1	2	1	2	1	1	1	1	2	2	1	1	2	1	1	1	1	2	1	1	1	1	1	1	2	1	1	1	2	2	1
Haplo_326	0%	0.22%	0.63%	0.31%	0%	0%	0%	1	1	1	1	1	1	1	1	1	1	1	1	1	1	1	1	1	1	1	1	1	1	2	1	1	1	1	1	1	1	1	1	1	1	1	1	1	1	1	1	1	1	2	1	1	1	1	1	1	1	2
Haplo_345	0%	0%	0.63%	0.61%	0%	0%	0%	1	1	1	1	1	1	1	2	1	1	1	1	1	1	1	1	1	1	1	1	1	1	1	1	1	1	1	1	1	1	1	1	1	1	1	1	1	1	1	1	1	1	2	1	1	1	1	1	1	1	2
Haplo_36	0%	0%	0.63%	0%	0%	0%	0%	1	1	1	2	2	2	2	1	1	1	2	2	1	2	1	1	1	1	2	2	1	1	2	1	2	1	2	2	1	1	1	1	2	1	1	1	1	1	1	1	1	1	1	1	2	1	1	1	2	2	1
Haplo_362	0%	0%	0.63%	0%	0.25%	0%	0%	1	1	1	1	2	1	1	1	1	1	1	1	1	1	1	1	1	1	2	1	1	2	2	1	1	1	1	1	1	1	1	1	1	1	1	1	1	1	1	1	1	1	2	1	1	1	1	1	1	1	2
Haplo_380	0%	0%	0.63%	0.61%	0.25%	0%	0%	1	1	1	1	2	1	1	1	2	1	1	1	1	1	1	1	1	1	2	1	1	2	1	2	1	1	1	1	1	1	1	1	2	1	1	1	1	1	1	1	1	1	1	1	2	1	1	2	1	1	2
Haplo_47	0%	0%	0.63%	2.15%	1.49%	1.79%	0.95%	1	1	1	2	2	2	2	1	1	1	2	2	1	2	1	1	1	1	2	2	1	1	2	2	1	1	1	1	1	1	1	2	1	1	1	1	1	1	1	1	1	2	1	1	1	1	1	1	2	2	1
Haplo_5	0%	0%	0.63%	0%	0.74%	0%	0%	1	1	1	1	2	2	2	1	1	1	2	2	1	2	1	1	1	1	2	2	1	1	2	2	1	1	1	1	2	2	1	1	2	1	1	1	1	2	1	1	1	1	1	1	2	1	1	1	2	2	1
Haplo_9	0%	0%	0.63%	0%	0%	0%	0%	1	1	1	2	1	1	1	1	1	1	1	1	1	1	1	1	1	1	1	1	1	1	1	2	1	1	1	1	1	1	1	1	1	1	1	1	1	2	1	2	1	1	2	1	1	2	1	1	1	2	2	1
Haplo_97	0%	3.45%	0.63%	0%	0%	0%	0%	1	1	2	1	1	1	1	1	1	1	1	1	1	1	1	1	1	1	1	1	1	1	1	1	1	1	1	1	1	1	1	1	1	1	1	1	1	1	1	1	1	1	2	1	1	1	1	1	1	1	2
Haplo_100	0%	0%	0.32%	0.92%	0%	0%	0%	1	1	2	1	1	1	1	1	1	1	1	1	1	1	1	1	1	1	1	1	1	1	1	2	1	1	1	1	1	1	1	2	1	1	1	1	1	1	1	1	1	2	1	1	1	1	1	1	2	2	1
Haplo_137	2.34%	9.27%	0.32%	0.31%	2.72%	0%	0%	1	1	2	2	1	1	1	1	1	1	1	1	1	1	1	1	1	1	1	1	1	1	1	1	1	1	1	1	1	1	1	1	1	1	1	1	1	1	1	1	1	1	2	1	1	1	1	1	1	1	2
Haplo_145	0%	0%	0.32%	0%	0%	0%	0%	1	1	2	2	1	1	1	1	1	1	1	1	1	1	1	1	1	1	1	1	1	1	1	2	2	1	2	2	1	1	1	1	2	1	1	1	1	1	1	1	1	1	1	1	2	2	1	1	2	2	1
Haplo_19	0%	0%	0.32%	0%	0%	0%	0%	1	1	1	2	2	1	1	1	2	1	1	1	1	1	1	1	1	1	2	1	1	2	1	2	1	1	1	1	1	1	1	2	1	1	1	1	1	1	1	1	1	2	1	1	1	1	1	1	2	2	1
Haplo_200	0%	0.43%	0.32%	0%	0%	0%	0%	1	1	2	2	2	2	2	1	1	1	2	2	1	2	1	1	1	1	2	2	1	1	2	2	1	1	1	1	1	1	1	1	2	1	1	1	1	1	1	1	1	1	1	1	2	1	1	2	1	1	2
Haplo_216	4.69%	0.22%	0.32%	0%	1.49%	0%	0%	1	2	2	2	1	1	1	1	1	1	1	1	1	1	1	1	1	1	1	1	1	1	1	2	1	1	1	1	1	1	1	1	2	1	1	1	1	1	1	1	1	1	1	1	2	1	1	2	1	1	2
Haplo_218	0%	0%	0.32%	0%	0.25%	0%	0%	1	2	2	2	1	1	1	1	1	1	1	1	1	1	1	1	1	1	1	1	1	1	1	2	1	1	1	1	1	1	1	2	1	1	1	1	1	1	1	1	1	2	1	1	1	1	1	1	2	2	1
Haplo_219	0%	0%	0.32%	0%	0%	0%	0%	1	2	2	2	1	1	1	1	1	1	1	1	1	1	1	1	1	1	1	1	1	1	1	2	2	1	1	1	1	1	1	1	1	1	1	1	2	1	1	1	2	2	1	1	2	1	1	1	2	2	2
Haplo_235	0%	0%	0.32%	0%	0%	0%	0%	1	2	2	2	2	1	1	1	2	1	1	1	1	1	1	1	1	1	2	1	1	2	1	2	1	1	1	1	2	2	1	1	1	1	1	1	1	1	1	1	1	1	2	1	1	1	1	1	1	1	2
Haplo_249	0%	0%	0.32%	0%	0%	0%	0%	1	2	2	2	2	2	2	1	1	1	2	2	1	2	1	1	1	1	2	2	1	1	2	2	1	1	1	1	2	2	1	1	2	1	1	1	1	2	1	1	1	1	1	1	2	1	1	1	2	2	1
Haplo_280	0%	0%	0.32%	0%	0%	0%	0%	2	2	2	2	1	1	1	1	2	1	1	1	1	1	1	1	1	1	2	1	1	2	1	2	1	1	1	1	1	1	1	2	1	1	1	1	1	1	1	1	1	2	1	1	1	1	1	1	2	2	1
Haplo_29	0%	0%	0.32%	0%	0.25%	0%	0%	1	1	1	2	2	2	2	1	1	1	2	2	1	2	1	1	1	1	2	2	1	1	2	1	1	1	1	1	1	1	1	1	1	1	1	1	1	1	1	1	1	1	2	1	1	1	1	1	2	2	1
Haplo_292	0%	0%	0.32%	0%	0%	0%	0%	1	1	1	1	2	2	2	1	1	1	2	2	1	2	1	1	1	1	2	2	1	1	2	2	1	1	1	1	1	1	1	2	1	1	1	1	1	1	1	1	1	2	1	1	1	1	1	1	2	2	1
Haplo_3	0%	0%	0.32%	0%	0%	0%	0%	1	1	1	1	2	1	1	1	2	1	1	1	1	1	1	1	1	1	2	1	1	2	1	2	1	1	1	1	1	1	1	2	1	1	1	1	1	1	1	1	1	2	1	1	1	1	1	1	2	2	1
Haplo_315	0%	0%	0.32%	3.37%	0.74%	0%	0%	1	1	1	1	1	1	1	1	1	1	1	1	1	1	1	1	1	1	1	1	1	1	1	2	1	1	1	1	1	1	1	1	1	1	1	1	1	2	1	2	1	1	2	1	1	2	1	1	1	2	2	1
Haplo_317	0%	0%	0.32%	0%	0%	0%	0%	1	1	1	1	1	1	1	1	1	1	1	1	1	1	1	1	1	1	1	1	1	1	1	2	1	1	1	1	1	1	1	1	2	1	1	1	1	1	1	1	1	1	1	1	2	1	1	2	1	1	2
Haplo_324	0%	0%	0.32%	0%	0%	0%	0%	1	1	1	1	1	1	1	1	1	1	1	1	1	1	1	1	1	1	1	1	1	1	1	2	2	1	2	1	1	1	2	1	2	1	1	1	1	1	1	1	1	1	1	1	2	1	1	2	1	1	2
Haplo_328	0%	0%	0.32%	0%	0%	0%	0%	1	1	1	1	1	1	1	1	1	1	1	1	1	1	1	1	1	1	1	1	1	1	2	2	1	1	1	1	1	1	1	1	2	1	1	1	1	1	1	1	1	1	1	1	2	1	1	2	1	1	2
Haplo_348	0%	0%	0.32%	0.31%	0%	0%	0%	1	1	1	1	1	1	1	2	1	1	1	1	1	1	1	1	1	1	1	1	1	1	1	2	1	1	1	1	1	1	1	1	2	1	1	1	1	1	1	1	1	1	1	1	2	1	1	1	1	1	2
Haplo_349	0%	0%	0.32%	0.31%	0.50%	0%	0%	1	1	1	1	1	1	1	2	1	1	1	1	1	1	1	1	1	1	1	1	1	1	1	2	1	1	1	1	1	1	1	1	2	1	1	1	1	1	1	1	1	1	1	1	2	1	1	2	1	1	2
Haplo_35	0%	0%	0.32%	0%	0%	0%	0%	1	1	1	2	2	2	2	1	1	1	2	2	1	2	1	1	1	1	2	2	1	1	2	1	2	1	1	2	1	1	1	1	1	1	1	1	1	1	1	1	1	1	2	1	1	1	1	1	1	1	2
Haplo_383	0%	0%	0.32%	0%	0%	0%	0%	1	1	1	1	2	1	1	1	2	1	1	1	1	1	1	1	1	1	2	1	1	2	1	2	1	1	1	1	2	2	1	1	2	1	1	1	1	2	1	1	1	1	1	1	2	1	1	1	2	2	1
Haplo_387	0%	0%	0.32%	0%	0%	0%	0%	1	1	1	1	2	2	2	1	1	1	2	2	1	2	1	1	1	1	1	1	1	1	1	2	1	1	1	1	1	1	1	1	2	1	1	1	1	1	1	1	1	1	1	1	1	2	1	1	1	1	2
Haplo_389	0%	0%	0.32%	0%	0%	0%	0%	1	1	1	1	2	2	2	1	1	1	2	2	1	2	1	1	1	1	2	2	1	1	2	1	1	1	1	1	1	1	1	1	1	1	1	1	1	1	1	1	1	2	1	1	1	1	1	1	2	2	2

Haplotype nr	America	East Asia	Europe	Middle East	Central South Asia	Oceania	Subsaharian Africa	rs10265249	rs7458593	rs4723034	rs7384927	rs10225302	rs13438031	rs28371550	rs6954044	rs33995922	rs28371551	rs17159769	rs28371552	rs28371553	rs17159772	rs28371554	rs28371555	rs28371556	rs28371557	rs2302019	rs2302020	rs2302021	rs4988494	rs2267721	rs2267723	rs4988496	rs28371558	rs28371559	rs4988498	rs4988499	rs740336	rs28371560	rs2741	rs4988505	rs28371561	rs28371562	rs2228078	rs2586	rs28371563	rs28371564	rs28371565	rs28371566	rs6962369	rs10653361	rs4723039	rs28371567	rs28371568	rs28371569	rs28371570	rs11771444	rs11973385	rs11760522
Haplo_400	0%	0%	0.32%	0%	0.25%	0%	0%	1	1	1	1	2	2	2	1	1	1	2	2	1	2	1	1	2	1	2	2	1	1	2	1	1	1	1	1	1	1	1	1	1	1	1	1	1	1	1	1	1	1	2	1	1	1	1	1	1	1	2
Haplo_404	0%	0%	0.32%	0%	0%	0%	0%	1	1	1	1	2	2	2	1	1	1	2	2	1	2	1	1	2	1	2	2	1	1	2	2	1	1	1	1	1	1	1	1	2	1	1	1	1	1	1	1	1	1	1	1	2	1	1	2	1	2	2
Haplo_414	0%	0%	0.32%	0%	0%	0%	0%	1	1	1	2	1	1	1	2	1	1	1	1	1	1	1	1	1	1	1	1	1	1	1	2	1	1	1	1	1	1	1	1	2	1	1	1	1	1	1	1	1	1	1	1	2	1	1	2	1	1	2
Haplo_415	0%	0%	0.32%	0%	0%	0%	0%	1	1	1	2	2	1	1	1	1	1	1	1	1	1	1	1	1	1	2	1	1	2	2	1	1	1	1	1	1	1	1	1	1	1	1	1	1	1	1	1	1	1	2	1	1	1	1	1	1	1	2
Haplo_418	0%	0%	0.32%	0%	0%	0%	0%	1	1	1	2	2	2	2	1	1	1	2	2	1	2	1	1	1	1	2	2	1	1	2	1	1	1	1	1	1	1	1	1	1	1	1	1	1	1	1	1	1	1	2	1	1	1	1	1	1	1	2
Haplo_419	0%	0%	0.32%	0%	0%	0%	0%	1	1	1	2	2	2	2	1	1	1	2	2	1	2	1	1	1	1	2	2	1	1	2	2	1	1	1	1	1	1	1	1	2	1	1	1	1	1	1	1	1	1	1	1	2	1	1	2	1	1	2
Haplo_50	0%	0%	0.32%	2.15%	0.99%	0%	0.95%	1	1	1	2	2	2	2	1	1	1	2	2	1	2	1	1	1	1	2	2	1	1	2	2	2	1	2	2	1	1	1	1	2	1	1	1	1	1	1	1	1	1	1	1	2	2	1	1	2	2	1
Haplo_66	0%	0%	0.32%	0%	0%	0%	0%	1	2	1	2	2	2	2	1	1	1	2	2	1	2	1	1	1	1	2	2	1	1	2	2	2	1	2	2	1	1	1	1	2	1	1	1	1	1	1	1	1	1	1	1	2	2	1	1	2	2	1
Haplo_83	0%	0%	0.32%	0%	0.25%	0%	0%	1	1	1	1	2	1	1	1	2	1	1	1	1	1	1	1	1	1	2	1	1	2	1	2	1	1	1	1	1	1	1	2	1	1	1	1	1	1	1	1	1	2	1	1	1	1	1	1	2	2	1
Haplo_88	0%	0.22%	0.32%	0%	0%	1.79%	0%	1	1	1	1	1	1	1	1	1	1	1	1	1	1	1	1	1	1	1	1	1	1	1	1	1	1	1	1	1	1	1	1	1	1	1	1	1	1	1	1	1	1	2	1	1	1	1	1	1	1	2
Haplo_92	0%	0%	0.32%	0%	0%	0%	0%	1	1	1	1	2	2	2	1	1	1	2	2	1	2	1	1	2	1	2	2	1	1	2	1	1	1	1	1	1	1	1	1	1	1	1	1	1	1	1	1	1	1	2	1	1	1	1	1	1	1	2
Haplo_98	0%	0%	0.32%	0%	0.99%	0%	0%	1	1	2	1	1	1	1	1	1	1	1	1	1	1	1	1	1	1	1	1	1	1	1	2	1	1	1	1	1	1	1	1	2	1	1	1	1	1	1	1	1	1	1	1	2	1	1	2	1	1	2
Haplo_1	0%	0%	0%	0%	0.25%	0%	0%	1	1	1	1	1	1	1	1	1	1	1	1	1	1	1	1	1	1	1	1	1	1	1	1	1	1	1	1	1	1	1	1	1	1	1	1	1	1	1	1	1	1	2	1	1	1	1	1	1	1	2
Haplo_10	1.56%	0%	0%	0.92%	1.49%	0%	0%	1	1	1	2	1	1	1	1	1	1	1	1	1	1	1	1	1	1	1	1	1	1	1	2	1	1	1	1	1	1	1	1	2	1	1	1	1	1	1	1	1	1	1	1	2	1	1	2	1	1	2
Haplo_101	0%	0%	0%	0.31%	0%	0%	0%	1	1	2	1	1	1	1	1	1	1	1	1	1	1	1	1	1	1	1	1	1	1	1	2	2	1	2	2	1	1	1	1	2	1	1	1	1	1	1	1	1	1	1	1	2	2	1	1	2	2	1
Haplo_102	0%	0%	0%	0.31%	0%	0%	0%	1	1	2	1	1	1	1	1	1	1	1	1	1	1	1	1	1	1	2	2	1	1	2	1	1	1	1	1	1	1	1	1	1	1	1	1	1	1	1	1	1	1	2	1	1	1	1	1	1	1	2
Haplo_103	0%	0%	0%	0%	0%	0%	0.48%	1	1	2	1	2	1	1	1	1	1	1	1	1	1	1	1	1	1	2	1	1	2	1	1	1	1	1	1	1	1	1	1	1	1	1	1	1	1	1	1	1	1	2	1	1	1	1	1	2	2	1
Haplo_104	0%	0%	0%	0%	0%	0%	0.48%	1	1	2	1	2	1	1	1	1	1	1	1	1	1	1	1	1	1	2	1	1	2	1	2	1	1	1	1	1	1	1	1	2	1	1	1	1	1	1	1	1	1	1	1	2	1	1	1	2	2	1
Haplo_105	0%	0%	0%	0%	0%	0%	0.95%	1	1	2	1	2	1	1	1	1	1	1	1	1	1	1	1	1	1	2	1	1	2	1	2	1	1	1	1	1	1	1	1	2	1	1	1	1	1	1	1	1	1	1	1	2	1	1	2	1	1	2
Haplo_106	0%	0%	0%	0.31%	0%	0%	0.95%	1	1	2	1	2	1	1	1	1	1	1	1	1	1	1	1	1	1	2	1	1	2	2	2	1	1	1	1	1	1	1	1	2	1	1	1	1	1	1	1	1	1	1	1	2	1	1	2	1	1	2
Haplo_107	0%	0%	0%	0%	0%	0%	1.90%	1	1	2	1	2	1	1	1	1	1	1	1	1	1	1	1	1	1	2	1	1	2	2	2	1	1	1	1	1	1	1	1	2	1	1	1	1	1	1	1	1	1	1	1	2	1	2	1	2	2	1
Haplo_108	0%	0%	0%	0%	0%	0%	0.48%	1	1	2	1	2	1	1	1	1	1	1	1	1	1	1	1	1	1	2	1	1	2	2	2	1	1	1	1	1	1	1	1	2	1	1	1	1	2	1	1	1	1	1	1	2	1	1	1	2	2	1
Haplo_109	0%	0%	0%	0%	0%	0%	0.95%	1	1	2	1	2	1	1	1	1	1	1	1	1	1	1	1	1	1	2	1	1	2	2	2	2	1	1	1	1	1	1	1	1	1	1	1	1	1	1	1	1	1	1	1	1	1	1	1	2	2	1
Haplo_11	1.56%	0%	0%	0%	0%	0%	0%	1	1	1	2	1	1	1	1	1	1	1	1	1	1	1	1	1	1	1	1	1	1	1	2	1	1	1	1	1	1	1	2	1	1	1	1	1	1	1	1	1	2	1	1	1	1	1	1	1	1	2
Haplo_110	0%	0%	0%	0%	0%	0%	0.95%	1	1	2	1	2	1	1	1	1	1	1	1	1	1	1	1	1	1	2	1	1	2	2	2	2	1	2	2	1	1	1	1	2	1	1	1	1	1	1	1	1	2	1	1	2	1	1	2	2	2	1
Haplo_111	0%	0%	0%	0%	0.50%	0%	0%	1	1	2	1	2	1	1	1	1	1	1	2	1	1	1	1	1	1	2	1	2	2	2	2	1	1	1	1	1	1	1	1	2	1	1	1	1	1	1	1	1	2	1	1	2	1	1	2	2	1	1
Haplo_112	0%	0.86%	0%	0%	0%	0%	0%	1	1	2	1	2	1	1	1	1	1	1	2	1	1	1	1	1	1	2	1	2	2	2	2	1	1	1	1	1	1	1	2	1	1	1	1	1	1	1	1	1	2	1	1	1	1	1	1	2	2	1
Haplo_113	0%	0%	0%	0%	0%	0%	0.48%	1	1	2	1	2	1	1	1	1	1	1	2	1	1	1	1	1	1	2	1	2	2	2	2	1	1	1	1	2	2	1	1	2	1	1	1	1	2	1	1	1	1	1	1	2	1	1	1	2	2	1
Haplo_114	0%	0%	0%	0%	0%	0%	0.48%	1	1	2	1	2	1	1	1	1	1	1	2	1	1	1	1	1	1	2	1	2	2	2	2	2	1	1	1	1	1	1	1	1	1	1	1	2	1	1	1	2	2	1	1	2	1	1	1	2	2	2
Haplo_115	0%	0%	0%	0%	0%	0%	0.48%	1	1	2	1	2	1	1	1	1	1	1	2	1	1	1	1	1	1	2	1	2	2	2	2	2	1	2	2	1	1	1	1	2	1	1	1	1	1	1	1	1	1	1	1	2	2	1	1	2	2	1
Haplo_116	0%	0%	0%	0%	0%	0%	0.95%	1	1	2	1	2	1	1	1	2	1	1	1	1	1	1	1	1	1	2	1	1	2	1	1	1	1	1	1	1	1	1	1	1	1	1	1	1	1	1	1	1	1	1	1	1	1	1	1	2	2	1
Haplo_117	0%	0%	0%	0%	0%	0%	0.95%	1	1	2	1	2	1	1	1	2	1	1	1	1	1	1	1	1	1	2	1	1	2	1	2	1	1	1	1	1	1	1	1	2	1	1	1	1	1	1	1	1	1	1	1	2	1	1	2	1	1	2
Haplo_118	0%	0%	0%	0%	0%	0%	0.48%	1	1	2	1	2	1	1	1	2	1	1	1	1	1	1	1	1	1	2	1	1	2	1	2	1	1	1	1	1	1	1	2	1	1	1	1	1	1	1	1	1	2	1	1	1	1	1	1	2	2	1
Haplo_119	0%	0%	0%	0%	0%	0%	0.48%	1	1	2	1	2	1	1	1	2	1	1	1	1	1	1	1	1	1	2	1	1	2	2	1	1	1	1	1	1	1	1	1	1	1	1	1	1	1	1	1	1	1	1	1	1	1	1	1	2	2	1
Haplo_120	0%	0%	0%	0%	0%	0%	0.95%	1	1	2	1	2	2	1	1	1	1	2	2	1	1	1	1	1	1	2	2	1	1	2	1	2	1	2	2	1	1	1	1	2	1	1	1	1	1	1	1	1	1	1	1	2	1	1	1	2	2	1
Haplo_121	0%	0%	0%	0.31%	0%	0%	0%	1	1	2	1	2	2	1	1	1	1	2	2	1	1	1	1	1	1	2	2	1	1	2	2	1	1	1	1	1	1	1	1	1	1	1	1	1	1	1	1	1	1	1	1	2	1	1	1	2	2	1
Haplo_122	0%	0%	0%	0%	0%	0%	0.48%	1	1	2	1	2	2	1	1	1	1	2	2	1	1	1	1	1	1	2	2	1	1	2	2	1	1	1	1	2	2	1	1	2	1	1	1	1	2	1	1	1	1	1	1	2	1	1	1	2	2	1
Haplo_123	0%	0%	0%	0%	0%	0%	0.48%	1	1	2	1	2	2	2	1	1	1	1	1	1	1	1	1	1	1	2	2	1	1	2	2	2	1	1	1	1	1	1	1	1	1	1	1	2	1	1	1	2	2	1	1	2	1	1	1	2	2	2
Haplo_124	0%	0%	0%	0%	0%	0%	0.95%	1	1	2	1	2	2	2	1	1	1	1	1	1	1	1	2	1	1	2	2	1	1	2	2	1	1	1	1	1	1	1	1	2	1	1	1	1	2	1	1	1	1	1	1	2	1	1	1	2	2	1
Haplo_125	0%	0%	0%	0.61%	0%	0%	0%	1	1	2	1	2	2	2	1	1	1	1	1	1	1	1	2	1	1	2	2	1	1	2	2	2	1	1	1	1	1	1	1	1	1	1	1	1	1	1	1	1	1	1	1	1	1	1	1	2	2	1
Haplo_126	0%	0%	0%	0%	0%	0%	0.48%	1	1	2	1	2	2	2	1	1	1	2	2	1	2	1	1	1	1	2	2	1	1	2	2	1	1	1	1	1	1	1	1	2	1	1	1	1	1	1	1	1	1	1	1	2	1	1	1	1	1	2
Haplo_127	0%	0%	0%	0%	0%	0%	0.95%	1	1	2	1	2	2	2	1	1	1	2	2	1	2	1	1	1	1	2	2	1	1	2	2	1	1	1	1	1	1	1	1	2	1	1	1	1	1	1	1	1	1	1	1	2	1	1	1	2	2	1
Haplo_128	0%	0%	0%	0%	0%	0%	1.43%	1	1	2	1	2	2	2	1	1	1	2	2	1	2	1	1	1	1	2	2	1	1	2	2	1	1	1	1	1	1	1	1	2	1	1	1	1	1	1	1	1	1	1	1	2	1	1	2	1	1	2
Haplo_129	0%	0%	0%	0%	0.25%	0%	0%	1	1	2	1	2	2	2	1	1	1	2	2	1	2	1	1	1	1	2	2	1	1	2	2	1	1	1	1	1	1	1	1	2	1	1	1	1	1	1	1	1	2	1	1	2	1	1	1	1	1	1
Haplo_130	0%	0%	0%	0%	0%	0%	0.48%	1	1	2	1	2	2	2	1	1	1	2	2	1	2	1	1	1	1	2	2	1	1	2	2	1	1	1	1	1	1	1	1	2	1	1	1	1	2	1	1	1	1	1	1	2	1	1	1	2	2	1
Haplo_131	0%	0%	0%	0%	0%	0%	0.48%	1	1	2	1	2	2	2	1	1	1	2	2	1	2	1	1	1	1	2	2	1	1	2	2	1	1	1	1	1	1	1	2	1	1	1	1	1	1	1	1	1	2	1	1	1	1	1	1	2	2	1
Haplo_132	0%	0%	0%	0%	0%	0%	0.95%	1	1	2	1	2	2	2	1	1	1	2	2	1	2	1	1	1	1	2	2	1	1	2	2	1	1	1	1	2	2	1	1	2	1	1	1	1	2	1	1	1	1	1	1	2	1	1	1	2	2	1
Haplo_133	0%	0%	0%	0%	0%	0%	0.95%	1	1	2	1	2	2	2	1	1	1	2	2	1	2	1	1	1	1	2	2	1	1	2	2	2	1	1	1	1	1	1	1	1	1	1	1	1	1	1	1	1	1	1	1	1	1	1	1	2	2	1
Haplo_134	0%	0%	0%	0%	0%	0%	0.95%	1	1	2	1	2	2	2	1	1	1	2	2	1	2	1	1	1	1	2	2	1	1	2	2	2	1	1	1	1	1	1	1	1	1	1	1	2	1	1	1	2	2	1	1	2	1	1	1	2	2	2
Haplo_135	0%	0%	0%	0%	0%	0%	0.48%	1	1	2	1	2	2	2	1	1	1	2	2	1	2	1	1	1	1	2	2	1	1	2	2	2	1	2	2	1	1	1	1	2	1	1	1	1	1	1	1	1	1	1	1	2	2	1	1	2	2	1
Haplo_136	0%	0%	0%	0%	0%	0%	0.95%	1	1	2	1	2	2	2	1	1	1	2	2	1	2	1	1	1	1	2	2	1	1	2	2	2	1	2	2	1	1	1	1	2	1	1	1	1	1	1	1	1	2	1	1	2	1	1	2	2	2	1
Haplo_138	2.34%	0%	0%	0%	0%	0%	0%	1	1	2	2	1	1	1	1	1	1	1	1	1	1	1	1	1	1	1	1	1	1	1	1	1	1	1	1	1	1	1	1	1	1	1	1	1	2	1	1	1	1	1	1	2	2	1	1	1	1	1	1	2
Haplo_139	0%	0.22%	0%	0%	0%	0%	0%	1	1	2	2	1	1	1	1	1	1	1	1	1	1	1	1	1	1	1	1	1	1	1	1	1	1	1	1	1	1	1	2	1	1	1	1	1	1	1	1	1	2	1	1	1	1	1	1	2	2	1
Haplo_140	0%	0%	0%	0%	0.74%	0%	0%	1	1	2	2	1	1	1	1	1	1	1	1	1	1	1	1	1	1	1	1	1	1	1	2	1	1	1	1	1	1	1	1	1	1	1	1	1	1	1	1	1	1	2	1	1	1	1	1	1	1	2
Haplo_141	0%	0.43%	0%	0%	0%	0%	0%	1	1	2	2	1	1	1	1	1	1	1	1	1	1	1	1	1	1	1	1	1	1	1	2	1	1	1	1	1	1	1	1	1	1	1	1	1	1	1	1	1	2	1	1	1	1	1	1	2	2	1
Haplo_142	0%	0.43%	0%	0%	0.25%	0%	0%	1	1	2	2	1	1	1	1	1	1	1	1	1	1	1	1	1	1	1	1	1	1	1	2	1	1	1	1	1	1	1	1	2	1	1	1	1	1	1	1	1	1	1	1	2	1	1	2	1	1	2
Haplo_143	0%	0%	0%	0%	0.25%	0%	0%	1	1	2	2	1	1	1	1	1	1	1	1	1	1	1	1	1	1	1	1	1	1	1	2	1	1	1	1	1	1	1	1	2	1	1	1	1	2	1	1	1	1	1	1	2	1	1	1	2	2	1
Haplo_144	0%	0.43%	0%	0%	0.25%	0%	0%	1	1	2	2	1	1	1	1	1	1	1	1	1	1	1	1	1	1	1	1	1	1	1	2	1	1	1	1	1	1	1	2	1	1	1	1	1	1	1	1	1	2	1	1	1	1	1	1	2	2	1
Haplo_146	0%	0.86%	0%	0%	0%	0%	0%	1	1	2	2	1	1	1	1	1	1	1	1	1	1	1	1	1	1	1	1	1	1	2	1	1	1	1	1	1	1	1	1	1	1	1	1	1	1	1	1	1	1	2	1	1	1	1	1	1	1	2
Haplo_147	0%	0.22%	0%	0%	0%	0%	0%	1	1	2	2	1	1	1	1	1	1	1	2	1	1	1	1	1	1	2	1	2	2	2	2	1	1	1	1	1	1	1	2	1	1	1	1	1	1	1	1	1	2	1	1	1	1	1	1	1	1	2
Haplo_148	0%	0%	0%	0%	0%	0%	0.48%	1	1	2	2	2	1	1	1	1	1	1	1	1	1	1	1	1	1	1	1	1	1	1	2	1	1	1	1	2	2	1	1	2	1	1	1	1	2	1	1	1	1	1	1	2	1	1	1	2	2	1
Haplo_149	0%	0%	0%	0%	0%	0%	0.48%	1	1	2	2	2	1	1	1	1	1	1	1	1	1	1	1	1	1	2	1	1	2	1	1	1	1	1	1	1	1	1	1	1	1	1	1	1	1	1	1	1	1	1	1	1	1	1	1	2	2	1
Haplo_150	0%	0%	0%	0%	0%	0%	0.48%	1	1	2	2	2	1	1	1	1	1	1	1	1	1	1	1	1	1	2	1	1	2	1	2	1	1	1	1	1	1	1	1	2	1	1	1	1	1	1	1	1	1	1	1	2	1	2	1	2	2	1
Haplo_151	0%	0%	0%	0%	0%	0%	1.43%	1	1	2	2	2	1	1	1	1	1	1	1	1	1	1	1	1	1	2	1	1	2	1	2	1	1	1	1	1	1	1	2	1	1	1	1	1	1	1	1	1	2	1	1	1	1	1	1	2	2	1
Haplo_152	0%	0%	0%	0%	0%	0%	0.48%	1	1	2	2	2	1	1	1	1	1	1	1	1	1	1	1	1	1	2	1	1	2	2	1	1	1	1	1	1	1	1	1	2	1	1	1	2	1	1	1	1	1	1	1	2	1	1	1	2	2	1
Haplo_153	0%	0%	0%	0%	0%	0%	0.48%	1	1	2	2	2	1	1	1	1	1	1	1	1	1	1	1	1	1	2	1	1	2	2	1	2	1	2	2	1	1	1	1	2	1	1	1	1	1	1	1	1	1	1	1	2	1	1	1	2	2	1

Haplotype nr	America	East Asia	Europe	Middle East	Central South Asia	Oceania	Subsaharian Africa	rs10265249	rs7458593	rs4723034	rs7384927	rs10225302	rs13438031	rs28371550	rs6954044	rs33995922	rs28371551	rs17159769	rs28371552	rs28371553	rs17159772	rs28371554	rs28371555	rs28371556	rs28371557	rs2302019	rs2302020	rs2302021	rs4988494	rs2267721	rs2267723	rs4988496	rs28371558	rs28371559	rs4988498	rs4988499	rs740336	rs28371560	rs2741	rs4988505	rs28371561	rs28371562	rs2228078	rs2586	rs28371563	rs28371564	rs28371565	rs28371566	rs6962369	rs10653361	rs4723039	rs28371567	rs28371568	rs28371569	rs28371570	rs11771444	rs11973385	rs11760522
Haplo_154	0%	0%	0%	0%	0%	0%	1.43%	1	1	2	2	2	1	1	1	1	1	1	1	1	1	1	1	1	1	2	1	1	2	2	2	1	1	1	1	1	1	1	1	2	1	1	1	1	1	1	1	1	1	1	1	2	1	1	2	1	1	2
Haplo_155	0%	0%	0%	0%	0%	0%	0.48%	1	1	2	2	2	1	1	1	1	1	1	1	1	1	1	1	1	1	2	1	1	2	2	2	1	1	1	1	1	1	1	1	2	1	1	1	1	1	1	1	1	1	1	1	2	1	2	1	2	2	1
Haplo_156	0%	0%	0%	0%	0%	0%	0.48%	1	1	2	2	2	1	1	1	1	1	1	1	1	1	1	1	1	1	2	1	1	2	2	2	1	1	1	1	1	1	1	1	2	1	1	1	1	2	1	1	1	1	1	1	2	1	1	1	2	2	1
Haplo_157	0%	0%	0%	0.31%	0%	0%	0%	1	1	2	2	2	1	1	1	1	1	1	1	1	1	1	1	1	1	2	1	1	2	2	2	1	1	1	1	2	2	1	1	2	1	1	1	1	2	1	1	1	1	1	1	2	1	1	1	2	2	1
Haplo_158	0%	4.31%	0%	0.61%	1.24%	1.79%	0%	1	1	2	2	2	1	1	1	1	1	1	2	1	1	1	1	1	1	2	1	2	2	2	1	1	1	1	1	1	1	1	1	1	1	1	1	1	1	1	1	1	1	2	1	1	1	1	1	1	1	2
Haplo_159	0%	0%	0%	0%	0%	0%	1.43%	1	1	2	2	2	1	1	1	1	1	1	2	1	1	1	1	1	1	2	1	2	2	2	1	1	1	1	1	1	1	1	1	1	1	1	1	1	1	1	2	1	1	2	1	1	1	1	1	2	2	1
Haplo_16	0%	0%	0%	0%	0%	3.57%	0%	1	1	1	2	2	1	1	1	1	1	1	1	1	1	1	1	1	1	2	1	1	2	2	1	1	1	1	1	1	1	1	1	1	1	1	1	1	1	1	1	1	1	2	1	1	1	1	1	1	1	2
Haplo_160	0.78%	0%	0%	0%	0%	0%	0%	1	1	2	2	2	1	1	1	1	1	1	2	1	1	1	1	1	1	2	1	2	2	2	1	1	1	1	1	1	1	1	1	1	1	1	2	1	1	1	1	1	1	2	2	1	1	1	1	1	1	2
Haplo_161	0%	0.22%	0%	0%	0%	0%	0%	1	1	2	2	2	1	1	1	1	1	1	2	1	1	1	1	1	1	2	1	2	2	2	1	2	1	2	1	1	1	2	1	1	1	1	1	1	1	1	1	1	1	2	1	1	1	1	1	1	1	2
Haplo_162	0%	0.86%	0%	0%	0%	0%	0%	1	1	2	2	2	1	1	1	1	1	1	2	1	1	1	1	1	1	2	1	2	2	2	2	1	1	1	1	1	1	1	1	1	1	1	1	1	1	1	1	1	1	2	1	1	1	1	1	1	1	2
Haplo_163	0%	0%	0%	0%	0%	0%	1.90%	1	1	2	2	2	1	1	1	1	1	1	2	1	1	1	1	1	1	2	1	2	2	2	2	1	1	1	1	1	1	1	1	1	1	1	1	1	1	1	1	1	2	1	1	1	1	1	1	2	2	2
Haplo_164	0%	0%	0%	0%	0%	0%	0.48%	1	1	2	2	2	1	1	1	1	1	1	2	1	1	1	1	1	1	2	1	2	2	2	2	1	1	1	1	1	1	1	1	1	1	1	1	2	1	2	1	1	2	1	1	2	1	1	1	2	2	1
Haplo_165	0%	0%	0%	0%	0%	0%	0.48%	1	1	2	2	2	1	1	1	1	1	1	2	1	1	1	1	1	1	2	1	2	2	2	2	1	1	1	1	1	1	1	1	2	1	1	1	1	1	1	1	1	1	1	1	2	1	1	1	1	1	2
Haplo_166	0%	0.22%	0%	0%	0%	0%	0%	1	1	2	2	2	1	1	1	1	1	1	2	1	1	1	1	1	1	2	1	2	2	2	2	1	1	1	1	1	1	1	1	2	1	1	1	1	1	1	1	1	2	1	1	2	1	1	2	2	1	1
Haplo_167	0%	0%	0%	0.31%	0%	0%	0.48%	1	1	2	2	2	1	1	1	1	1	1	2	1	1	1	1	1	1	2	1	2	2	2	2	1	1	1	1	1	1	1	1	2	1	1	1	1	2	1	1	1	1	1	1	2	1	1	1	2	2	1
Haplo_168	6.25%	3.66%	0%	0%	0%	0%	0%	1	1	2	2	2	1	1	1	1	1	1	2	1	1	1	1	1	1	2	1	2	2	2	2	1	1	1	1	1	1	2	1	1	1	1	1	1	1	1	1	1	2	1	1	1	1	1	1	1	1	2
Haplo_169	0%	3.66%	0%	0%	0%	0%	0.48%	1	1	2	2	2	1	1	1	1	1	1	2	1	1	1	1	1	1	2	1	2	2	2	2	1	1	1	1	1	1	1	2	1	1	1	1	1	1	1	1	1	2	1	1	1	1	1	1	2	2	1
Haplo_17	0%	0%	0%	0%	0.25%	0%	0%	1	1	1	2	2	1	1	1	1	1	1	1	1	1	1	1	1	1	2	1	1	2	2	2	1	1	1	1	1	1	1	2	1	1	1	1	1	1	1	1	1	2	1	1	1	1	1	1	2	2	1
Haplo_170	0%	0%	0%	0%	0.25%	0%	0%	1	1	2	2	2	1	1	1	1	1	1	2	1	1	1	1	1	1	2	1	2	2	2	2	1	1	1	1	2	2	1	1	2	1	1	1	1	2	1	1	1	1	1	1	2	1	1	1	2	2	1
Haplo_171	0%	0%	0%	0%	0%	0%	0.48%	1	1	2	2	2	1	1	1	1	1	1	2	1	1	1	1	1	1	2	1	2	2	2	2	2	1	1	1	1	1	1	1	1	1	1	1	1	1	1	1	1	1	1	1	1	1	1	1	2	2	1
Haplo_172	0%	0%	0%	0.31%	0%	0%	0.95%	1	1	2	2	2	1	1	1	1	1	1	2	1	1	1	1	1	1	2	1	2	2	2	2	2	1	2	2	1	1	1	1	2	1	1	1	1	1	1	1	1	1	1	1	2	2	1	1	2	2	1
Haplo_173	0%	0%	0%	0%	0%	0%	0.48%	1	1	2	2	2	1	1	1	1	1	1	2	1	1	1	1	1	1	2	1	2	2	2	2	2	1	2	2	1	1	1	1	2	1	1	1	1	1	1	1	1	1	1	1	2	2	1	1	2	2	2
Haplo_174	0%	0%	0%	0%	0.25%	0%	0%	1	1	2	2	2	1	1	1	1	1	1	2	1	1	1	2	1	1	2	1	2	2	2	1	1	1	1	1	1	1	1	1	1	1	1	1	1	1	1	1	1	1	2	1	1	1	1	1	1	1	2
Haplo_175	0%	0%	0%	0%	0.50%	0%	0%	1	1	2	2	2	1	1	1	2	1	1	1	1	1	1	1	1	1	2	1	1	2	1	2	1	1	1	1	1	1	1	1	1	1	1	1	2	1	2	1	1	2	1	1	2	1	1	1	2	2	1
Haplo_176	0%	0%	0%	0%	0%	0%	0.95%	1	1	2	2	2	2	1	1	1	1	2	2	1	1	1	1	1	1	2	2	1	1	2	2	1	1	1	1	1	1	1	1	1	1	1	1	1	2	1	1	1	1	1	1	2	1	1	1	2	2	1
Haplo_177	0%	0%	0%	0%	0%	0%	0.48%	1	1	2	2	2	2	1	1	1	1	2	2	1	1	1	1	1	1	2	2	1	1	2	2	1	1	1	1	1	1	1	2	1	1	1	1	1	1	1	1	1	2	1	1	1	1	1	1	2	2	1
Haplo_178	0%	0%	0%	0%	0%	0%	0.48%	1	1	2	2	2	2	1	1	1	2	2	2	1	1	1	1	1	2	2	2	1	1	2	2	1	1	1	1	1	1	1	1	2	1	1	1	1	1	1	1	1	1	1	1	2	1	2	1	2	2	1
Haplo_179	0%	0%	0%	0%	0%	0%	0.48%	1	1	2	2	2	2	1	1	1	2	2	2	1	1	1	1	1	2	2	2	1	1	2	2	2	1	1	1	1	1	1	1	1	1	1	1	1	1	1	1	1	1	1	1	1	1	1	1	2	2	1
Haplo_18	0%	0%	0%	0%	0.25%	0%	0%	1	1	1	2	2	1	1	1	1	1	1	2	1	1	1	1	1	1	2	1	2	2	2	2	1	1	1	1	1	1	1	1	2	1	1	1	1	1	1	1	1	2	1	1	2	1	1	2	2	1	1
Haplo_180	0%	0%	0%	0%	0%	0%	4.29%	1	1	2	2	2	2	1	1	1	2	2	2	2	1	1	1	1	1	2	1	1	1	2	2	1	1	1	1	1	1	1	1	2	1	1	1	1	1	1	1	1	1	1	1	2	1	1	1	1	1	2
Haplo_181	0%	0%	0%	0%	0%	0%	0.48%	1	1	2	2	2	2	1	1	1	2	2	2	2	1	1	1	1	1	2	1	1	1	2	2	2	1	1	1	1	1	1	1	1	1	1	1	1	1	1	1	1	1	1	1	1	1	1	1	2	2	1
Haplo_182	0%	0%	0%	0%	0%	0%	0.48%	1	1	2	2	2	2	2	1	1	1	1	1	1	1	1	1	1	1	2	1	1	2	1	1	1	1	1	1	1	1	1	1	1	1	1	1	1	1	1	1	1	1	2	1	1	1	1	1	1	1	2
Haplo_183	0%	0%	0%	0%	0%	0%	0.95%	1	1	2	2	2	2	2	1	1	1	1	1	1	1	1	1	1	1	2	1	1	2	1	2	1	1	1	1	1	1	1	1	2	1	1	1	1	1	1	1	1	1	1	1	2	1	1	1	2	2	1
Haplo_184	0%	0%	0%	0%	0%	0%	1.90%	1	1	2	2	2	2	2	1	1	1	1	1	1	1	1	2	1	1	2	2	1	1	2	2	1	1	1	1	1	1	1	1	1	1	1	1	2	1	2	1	1	2	1	1	2	1	1	1	2	2	1
Haplo_185	0%	0%	0%	0%	0%	0%	0.48%	1	1	2	2	2	2	2	1	1	1	1	1	1	1	1	2	1	1	2	2	1	1	2	2	1	1	1	1	1	1	1	1	2	1	1	1	1	1	1	1	1	1	1	1	2	1	1	2	1	1	2
Haplo_186	0%	0%	0%	0%	0%	0%	0.95%	1	1	2	2	2	2	2	1	1	1	1	1	1	1	1	2	1	1	2	2	1	1	2	2	1	1	1	1	1	1	1	1	2	1	1	1	1	2	1	1	1	1	1	1	2	1	1	1	2	2	1
Haplo_187	0%	0%	0%	0.31%	0%	0%	3.81%	1	1	2	2	2	2	2	1	1	1	1	1	1	1	1	2	1	1	2	2	1	1	2	2	2	1	1	1	1	1	1	1	1	1	1	1	1	1	1	1	1	1	1	1	1	1	1	1	2	2	1
Haplo_188	0%	0%	0%	0%	0%	0%	0.48%	1	1	2	2	2	2	2	1	1	1	1	1	1	1	1	2	1	1	2	2	1	1	2	2	2	1	1	1	1	1	1	1	1	1	1	1	1	1	1	1	1	1	2	1	1	1	1	1	2	2	1
Haplo_189	0%	0%	0%	0%	0%	0%	1.90%	1	1	2	2	2	2	2	1	1	1	1	1	1	1	1	2	1	1	2	2	1	1	2	2	2	1	1	1	1	1	1	1	1	1	1	1	2	1	1	1	2	2	1	1	2	1	1	1	2	2	1
Haplo_190	0%	0%	0%	0%	0%	0%	0.48%	1	1	2	2	2	2	2	1	1	1	1	1	1	1	1	2	1	1	2	2	1	1	2	2	2	1	1	1	1	1	1	1	1	1	1	1	2	1	1	1	2	2	1	1	2	1	1	1	2	2	2
Haplo_191	0%	0%	0%	0%	0%	0%	0.48%	1	1	2	2	2	2	2	1	1	1	1	1	1	1	1	2	1	1	2	2	1	1	2	2	2	1	2	2	1	1	1	1	2	1	1	1	1	1	1	1	1	1	1	1	2	1	1	1	2	2	1
Haplo_192	0%	0%	0%	0.31%	0%	0%	0.48%	1	1	2	2	2	2	2	1	1	1	1	1	1	1	1	2	1	1	2	2	1	1	2	2	2	1	2	2	1	1	1	1	2	1	1	1	1	1	1	1	1	1	1	1	2	2	1	1	2	2	1
Haplo_193	0%	0%	0%	0%	0%	0%	0.48%	1	1	2	2	2	2	2	1	1	1	2	2	1	1	1	1	1	1	2	2	1	1	2	2	1	1	1	1	1	1	1	1	1	1	1	1	2	1	1	1	1	2	1	1	1	1	1	1	2	2	1
Haplo_194	0%	0%	0%	0%	0%	0%	1.43%	1	1	2	2	2	2	2	1	1	1	2	2	1	1	1	1	1	1	2	2	1	1	2	2	2	1	1	1	1	1	1	1	1	1	1	1	2	1	1	1	2	2	1	1	2	1	1	1	2	2	1
Haplo_195	0%	0%	0%	0%	0%	0%	0.95%	1	1	2	2	2	2	2	1	1	1	2	2	1	2	1	1	1	1	2	2	1	1	2	1	1	1	1	1	1	1	1	1	1	1	1	1	1	1	1	1	1	1	1	1	1	1	1	1	2	2	1
Haplo_196	0%	0.22%	0%	0%	0%	0%	0%	1	1	2	2	2	2	2	1	1	1	2	2	1	2	1	1	1	1	2	2	1	1	2	1	1	1	1	1	1	1	1	1	1	1	1	1	1	1	1	1	1	1	2	1	1	1	1	1	1	1	2
Haplo_197	0%	0%	0%	0%	0.25%	0%	0%	1	1	2	2	2	2	2	1	1	1	2	2	1	2	1	1	1	1	2	2	1	1	2	1	1	1	1	1	1	1	1	1	1	1	1	1	1	1	1	1	1	1	2	1	1	1	1	1	2	2	1
Haplo_198	0%	0%	0%	0%	0%	0%	0.48%	1	1	2	2	2	2	2	1	1	1	2	2	1	2	1	1	1	1	2	2	1	1	2	1	1	1	1	1	1	1	1	1	1	1	1	1	1	1	1	2	1	1	2	1	1	1	1	1	2	2	1
Haplo_199	0%	0%	0%	0%	0%	0%	0.48%	1	1	2	2	2	2	2	1	1	1	2	2	1	2	1	1	1	1	2	2	1	1	2	1	2	1	1	1	1	1	1	1	1	1	1	1	1	1	1	1	1	1	1	1	1	1	1	1	2	2	1
Haplo_2	0%	0%	0%	0%	0.25%	0%	0%	1	1	1	1	2	1	1	1	2	1	1	1	1	1	1	1	1	1	2	1	1	2	1	1	1	1	1	1	1	1	1	1	1	1	1	1	1	1	1	1	1	1	2	1	1	1	1	1	1	1	2
Haplo_20	0%	0%	0%	1.23%	0%	0%	0%	1	1	1	2	2	1	1	1	2	1	1	1	1	1	1	1	1	1	2	1	1	2	1	2	1	1	1	1	2	2	1	1	2	1	1	1	1	2	1	1	1	1	1	1	2	1	1	1	2	2	1
Haplo_201	0%	0%	0%	0%	0%	0%	0.48%	1	1	2	2	2	2	2	1	1	1	2	2	1	2	1	1	1	1	2	2	1	1	2	2	1	1	1	1	1	1	1	1	2	1	1	1	1	1	1	1	1	1	1	1	2	1	2	1	2	2	1
Haplo_202	0%	0%	0%	0%	0%	0%	0.48%	1	1	2	2	2	2	2	1	1	1	2	2	1	2	1	1	1	1	2	2	1	1	2	2	1	1	1	1	1	1	1	1	2	1	1	1	1	1	1	1	1	2	1	1	2	1	1	1	2	2	1
Haplo_203	0%	1.08%	0%	0.31%	0.25%	0%	0%	1	1	2	2	2	2	2	1	1	1	2	2	1	2	1	1	1	1	2	2	1	1	2	2	1	1	1	1	1	1	1	2	1	1	1	1	1	1	1	1	1	2	1	1	1	1	1	1	2	2	1
Haplo_204	0%	0%	0%	0%	0%	0%	0.48%	1	1	2	2	2	2	2	1	1	1	2	2	1	2	1	1	1	1	2	2	1	1	2	2	2	1	1	1	1	1	1	1	1	1	1	1	1	1	1	1	1	1	1	1	1	1	1	1	2	2	1
Haplo_205	0%	0%	0%	0.31%	0%	0%	0.48%	1	1	2	2	2	2	2	1	1	1	2	2	1	2	1	1	1	1	2	2	1	1	2	2	2	1	2	2	1	1	1	1	2	1	1	1	1	1	1	1	1	1	1	1	2	2	1	1	2	2	1
Haplo_206	0%	0.22%	0%	0%	0%	0%	0%	1	1	2	2	2	2	2	1	1	1	2	2	1	2	1	1	2	1	2	2	1	1	2	2	1	1	1	1	1	1	1	1	2	1	1	1	1	1	1	1	1	1	1	1	2	1	1	2	1	1	2
Haplo_207	0%	0.86%	0%	0%	0%	0%	0%	1	2	2	1	1	1	1	1	1	1	1	1	1	1	1	1	1	1	1	1	1	1	1	1	1	1	1	1	1	1	1	1	1	1	1	1	1	1	1	1	1	1	2	1	1	1	1	1	1	1	2
Haplo_208	0%	0.22%	0%	0%	0%	0%	0%	1	2	2	1	2	1	1	1	1	1	1	2	1	1	1	1	1	1	2	1	2	2	2	2	1	1	1	1	1	1	1	2	1	1	1	1	1	1	1	1	1	2	1	1	1	1	1	1	1	1	2
Haplo_209	0%	0.22%	0%	0%	0%	0%	0%	1	2	2	1	2	1	1	1	2	1	1	1	1	1	1	1	1	1	2	1	1	2	1	1	1	1	1	1	1	1	1	1	1	1	1	1	1	1	1	1	1	1	2	1	1	1	1	1	1	1	2
Haplo_21	0%	0%	0%	0%	0%	0%	0.48%	1	1	1	2	2	2	1	1	1	2	2	2	1	1	1	1	1	2	2	2	1	1	2	2	1	1	1	1	1	1	1	1	2	1	1	1	1	1	1	1	1	1	1	1	2	1	2	1	2	2	1
Haplo_210	0%	0%	0%	0%	0.99%	0%	0%	1	2	2	1	2	1	1	1	2	1	1	1	1	1	1	1	1	1	2	1	1	2	1	2	1	1	1	1	1	1	1	1	1	1	1	1	2	1	2	1	1	2	1	1	2	1	1	1	2	2	1
Haplo_211	0%	0%	0%	0%	0.25%	0%	0%	1	2	2	1	2	2	2	1	1	1	2	2	1	2	1	1	1	1	2	2	1	1	2	1	1	1	1	1	1	1	1	1	1	1	1	1	1	1	1	1	1	1	2	1	1	1	1	1	1	1	2
Haplo_213	25%	0%	0%	0%	0.99%	0%	0%	1	2	2	2	1	1	1	1	1	1	1	1	1	1	1	1	1	1	1	1	1	1	1	1	1	1	1	1	1	1	1	1	1	1	1	2	1	1	1	1	1	1	2	2	1	1	1	1	1	1	2
Haplo_214	4.69%	0%	0%	0%	0%	0%	0%	1	2	2	2	1	1	1	1	1	1	1	1	1	1	1	1	1	1	1	1	1	1	1	1	2	1	2	1	1	1	2	1	1	1	1	1	1	1	1	1	1	2	1	1	1	1	1	1	2	2	2
Haplo_215	0%	0.86%	0%	0%	0%	0%	0%	1	2	2	2	1	1	1	1	1	1	1	1	1	1	1	1	1	1	1	1	1	1	1	2	1	1	1	1	1	1	1	1	1	1	1	1	1	1	1	1	1	1	2	1	1	1	1	1	1	1	2

Haplotype nr	America	East Asia	Europe	Middle East	Central South Asia	Oceania	Subsaharian Africa	rs10265249	rs7458593	rs4723034	rs7384927	rs10225302	rs13438031	rs28371550	rs6954044	rs33995922	rs28371551	rs17159769	rs28371552	rs28371553	rs17159772	rs28371554	rs28371555	rs28371556	rs28371557	rs2302019	rs2302020
Haplo_217	3.13%	0.22%	0%	0%	0%	0%	0%	1	2	2	2	1	1	1	1	1	1	1	1	1	1	1	1	1	1	1	1
Haplo_22	0%	0%	0%	0%	0.25%	0%	0%	1	1	1	2	2	2	2	1	1	1	2	1	1	2	1	1	1	1	2	2
Haplo_220	0%	0%	0%	0%	0.50%	0%	0%	1	2	2	2	1	1	1	1	1	1	1	1	1	1	1	1	1	1	1	1
Haplo_221	0%	0.22%	0%	0%	0%	0%	0%	1	2	2	2	1	1	1	1	1	1	1	2	1	1	1	1	1	1	2	1
Haplo_222	0%	0.22%	0%	0%	0%	0%	0%	1	2	2	2	1	1	1	2	1	1	1	1	1	1	1	1	1	1	1	1
Haplo_223	0%	0.22%	0%	0%	0%	0%	0%	1	2	2	2	2	1	1	1	1	1	1	1	1	1	1	1	1	1	1	1
Haplo_224	0%	0%	0%	0%	0%	0%	0.48%	1	2	2	2	2	1	1	1	1	1	1	1	1	1	1	1	1	1	2	1
Haplo_225	0.78%	0%	0%	0%	0.74%	0%	0%	1	2	2	2	2	1	1	1	1	1	1	2	1	1	1	1	1	1	2	1
Haplo_226	0.78%	0%	0%	0%	0%	0%	0%	1	2	2	2	2	1	1	1	1	1	1	2	1	1	1	1	1	1	2	1
Haplo_227	0%	0%	0%	0%	0%	0%	0.95%	1	2	2	2	2	1	1	1	1	1	1	2	1	1	1	1	1	1	2	1
Haplo_228	0%	0%	0%	0%	0.25%	0%	0%	1	2	2	2	2	1	1	1	1	1	1	2	1	1	1	1	1	1	2	1
Haplo_229	0%	0%	0%	0%	0.99%	0%	0%	1	2	2	2	2	1	1	1	1	1	1	2	1	1	1	1	1	1	2	1
Haplo_23	0%	0%	0%	0%	0.50%	0%	0%	1	1	1	2	2	2	2	1	1	1	2	1	1	2	1	1	1	1	2	2
Haplo_231	0%	0%	0%	0%	0.25%	0%	0%	1	2	2	2	2	1	1	1	2	1	1	1	1	1	1	1	1	1	2	1
Haplo_232	0%	0.65%	0%	0%	0%	0%	0%	1	2	2	2	2	1	1	1	2	1	1	1	1	1	1	1	1	1	2	1
Haplo_233	0%	0%	0%	0%	0.50%	0%	0%	1	2	2	2	2	1	1	1	2	1	1	1	1	1	1	1	1	1	2	1
Haplo_234	0%	0%	0%	0.31%	0%	0%	0%	1	2	2	2	2	1	1	1	2	1	1	1	1	1	1	1	1	1	2	1
Haplo_237	0%	0%	0%	0%	0.50%	0%	0%	1	2	2	2	2	1	1	1	2	1	1	1	1	1	1	1	1	1	2	1
Haplo_238	0%	0%	0%	0%	0.25%	0%	0%	1	2	2	2	2	1	1	1	2	1	1	1	1	1	1	1	1	1	2	1
Haplo_239	0%	0%	0%	0%	0%	0%	0.48%	1	2	2	2	2	2	1	1	1	2	2	2	2	1	1	1	1	1	2	1
Haplo_24	0%	0%	0%	0.31%	0%	0%	0%	1	1	1	2	2	2	2	1	1	1	2	2	1	2	1	1	1	1	2	2
Haplo_240	0%	0%	0%	0%	0.25%	0%	0%	1	2	2	2	2	2	2	1	1	1	1	1	1	1	1	1	1	1	2	2
Haplo_242	0%	0%	0%	0.31%	0%	0%	0%	1	2	2	2	2	2	2	1	1	1	2	2	1	2	1	1	1	1	2	2
Haplo_243	0.78%	0%	0%	0%	0%	0%	0%	1	2	2	2	2	2	2	1	1	1	2	2	1	2	1	1	1	1	2	2
Haplo_244	0%	0%	0%	0.31%	0%	0%	0%	1	2	2	2	2	2	2	1	1	1	2	2	1	2	1	1	1	1	2	2
Haplo_246	0%	0%	0%	0%	0%	0%	0.95%	1	2	2	2	2	2	2	1	1	1	2	2	1	2	1	1	1	1	2	2
Haplo_247	0%	0%	0%	0%	0.25%	0%	0%	1	2	2	2	2	2	2	1	1	1	2	2	1	2	1	1	1	1	2	2
Haplo_248	0%	0%	0%	1.84%	0%	0%	1.43%	1	2	2	2	2	2	2	1	1	1	2	2	1	2	1	1	1	1	2	2
Haplo_25	0%	0%	0%	0%	0.74%	0%	0%	1	1	1	2	2	2	2	1	1	1	2	2	1	2	1	1	1	1	2	2
Haplo_250	0%	0%	0%	0%	0%	0%	0.48%	2	1	2	1	2	1	1	1	1	1	1	2	1	1	1	1	1	1	2	1
Haplo_251	0%	0.65%	0%	0%	0%	0%	0%	2	1	2	2	1	1	1	1	1	1	1	1	1	1	1	1	1	1	1	1
Haplo_252	0%	0%	0%	0%	0%	0%	0.48%	2	1	2	2	2	1	1	1	1	1	1	1	1	1	1	1	1	1	2	1
Haplo_253	0%	0%	0%	0%	0%	0%	0.48%	2	1	2	2	2	1	1	1	1	1	1	1	1	1	1	1	1	1	2	1
Haplo_254	0%	0%	0%	0%	0%	0%	0.48%	2	1	2	2	2	1	1	1	1	1	1	2	1	1	1	1	1	1	2	1
Haplo_255	0%	0%	0%	0%	0%	0%	0.48%	2	1	2	2	2	1	1	1	2	1	1	1	1	1	1	1	1	1	2	1
Haplo_256	0%	0%	0%	0%	0%	0%	0.48%	2	1	2	2	2	2	1	1	1	1	1	2	1	1	1	1	1	1	2	1
Haplo_257	0%	0%	0%	0%	0%	0%	0.48%	2	1	2	2	2	2	1	1	1	1	2	2	1	1	1	1	1	1	2	2
Haplo_258	0%	0%	0%	0.31%	0%	0%	0%	2	1	2	2	2	2	1	1	1	2	2	2	1	1	1	1	1	1	2	2
Haplo_259	0%	0%	0%	0%	0%	0%	0.48%	2	1	2	2	2	2	1	1	1	2	2	2	1	1	1	1	1	1	2	2
Haplo_26	0%	0%	0%	0%	0.50%	0%	0%	1	1	1	2	2	2	2	1	1	1	2	2	1	2	1	1	1	1	2	2
Haplo_260	0%	0%	0%	0%	0%	0%	0.48%	2	1	2	2	2	2	1	1	1	2	2	2	1	1	1	1	1	1	2	2
Haplo_261	0%	0%	0%	0%	0%	0%	2.38%	2	1	2	2	2	2	1	1	1	2	2	2	1	1	1	1	1	1	2	2
Haplo_262	0%	0%	0%	0.31%	0%	0%	0.48%	2	1	2	2	2	2	1	1	1	2	2	2	1	1	1	1	1	2	2	2
Haplo_263	0%	0%	0%	0%	0%	0%	3.81%	2	1	2	2	2	2	1	1	1	2	2	2	1	1	1	1	1	2	2	2
Haplo_264	0%	0%	0%	0%	0%	0%	0.95%	2	1	2	2	2	2	1	1	1	2	2	2	1	1	1	1	1	2	2	2
Haplo_265	0%	0%	0%	0.31%	0%	0%	0%	2	1	2	2	2	2	1	1	1	2	2	2	1	1	1	1	1	2	2	2
Haplo_266	0%	0%	0%	0%	0%	0%	0.95%	2	1	2	2	2	2	2	1	1	1	1	1	1	1	1	1	1	1	2	2
Haplo_267	0%	0%	0%	0%	0%	0%	0.48%	2	1	2	2	2	2	2	1	1	1	1	1	1	1	1	2	1	1	2	2
Haplo_268	0%	0%	0%	0%	0%	0%	0.48%	2	1	2	2	2	2	2	1	1	1	2	2	1	2	1	1	1	1	2	2
Haplo_269	0%	0%	0%	0%	0%	0%	0.48%	2	1	2	2	2	2	2	1	1	1	2	2	1	2	1	1	1	1	2	2
Haplo_27	0%	0%	0%	0.31%	0%	0%	0%	1	1	1	2	2	2	2	1	1	1	2	2	1	2	1	1	1	1	2	2
Haplo_270	0%	0%	0%	0.31%	0%	0%	0%	2	1	2	2	2	2	2	1	1	1	2	2	1	2	1	1	1	1	2	2
Haplo_271	0%	0%	0%	0%	0%	0%	0.48%	2	1	2	2	2	2	2	1	1	1	2	2	1	2	1	1	1	1	2	2
Haplo_272	0%	0%	0%	0%	0%	0%	0.48%	2	1	2	2	2	2	2	1	1	1	2	2	1	2	1	1	1	1	2	2
Haplo_273	0%	0%	0%	0%	0%	0%	0.95%	2	1	2	2	2	2	2	1	1	1	2	2	1	2	1	1	1	1	2	2
Haplo_274	0%	0%	0%	0%	0%	0%	0.48%	2	1	2	2	2	2	2	1	1	1	2	2	1	2	1	1	1	1	2	2
Haplo_275	0%	0%	0%	0%	0%	0%	1.90%	2	1	2	2	2	2	2	1	1	1	2	2	1	2	1	1	1	1	2	2
Haplo_276	0%	0%	0%	0%	0%	0%	0.48%	2	1	2	2	2	2	2	1	1	1	2	2	1	2	1	1	1	1	2	2
Haplo_277	0%	0.22%	0%	0%	0.25%	0%	0%	2	2	2	2	1	1	1	1	1	1	1	1	1	1	1	1	1	1	1	1
Haplo_279	0%	0%	0%	0.31%	0%	0%	0%	2	2	2	2	1	1	1	1	1	1	1	1	1	1	1	1	1	1	1	1
Haplo_282	0%	0%	0%	0.61%	0.25%	0%	0%	2	2	2	2	1	1	1	2	1	1	1	1	1	1	1	1	1	1	1	1
Haplo_283	0%	0%	0%	0%	0.50%	0%	0%	2	2	2	2	1	1	1	2	1	1	1	1	1	1	1	1	1	1	1	1
Haplo_284	0%	0%	0%	0.31%	0%	0%	0%	2	2	2	2	1	1	1	2	1	1	1	1	1	1	1	1	1	1	1	1
Haplo_285	0%	0%	0%	0%	0.25%	0%	0%	2	2	2	2	1	1	1	2	1	1	1	1	1	1	1	1	1	1	1	1
Haplo_286	0%	0%	0%	0%	0.74%	0%	0%	2	2	2	2	2	1	1	1	2	1	1	1	1	1	1	1	1	1	2	1
Haplo_287	0%	0%	0%	0%	0.25%	0%	0%	2	2	2	2	2	1	1	1	2	1	1	1	1	1	1	1	1	1	2	1

Haplotype nr	rs2302021	rs4988494	rs2267721	rs2267723	rs4988496	rs28371558	rs28371559	rs4988498	rs4988499	rs740336	rs28371560	rs2741	rs4988505	rs28371561	rs28371562	rs2228078	rs2586	rs28371563	rs28371564	rs28371565	rs28371566	rs6962369	rs10653361	rs4723039	rs28371567	rs28371568	rs28371569	rs28371570	rs11771444	rs11973385	rs11760522
Haplo_217	1	1	1	2	1	1	1	1	1	1	1	2	1	1	1	1	1	1	1	1	1	2	1	1	1	1	1	1	1	1	2
Haplo_22	1	1	2	2	1	1	1	1	1	1	1	1	2	1	1	1	1	1	1	1	1	1	1	1	2	1	1	2	1	1	2
Haplo_220	1	1	1	2	2	1	2	2	1	1	1	1	2	1	1	1	1	1	1	1	1	1	1	1	2	2	1	1	2	2	1
Haplo_221	2	2	1	1	1	1	1	1	1	1	1	1	1	1	1	1	1	1	1	1	1	1	2	1	1	1	1	1	1	1	2
Haplo_222	1	1	1	1	1	1	1	1	1	1	1	1	1	1	1	1	1	1	1	1	1	1	2	1	1	1	1	1	1	1	2
Haplo_223	1	1	1	1	1	1	1	1	1	1	1	1	1	1	1	1	1	1	1	1	1	1	2	1	1	1	1	1	1	1	2
Haplo_224	1	2	1	2	1	1	1	1	1	1	1	1	2	1	1	1	1	1	1	1	1	1	1	1	2	1	2	1	2	2	1
Haplo_225	2	2	2	1	1	1	1	1	1	1	1	1	1	1	1	1	1	1	1	1	1	1	2	1	1	1	1	1	1	1	2
Haplo_226	2	2	2	1	1	1	1	1	1	1	1	1	1	1	1	2	1	1	1	1	1	1	2	2	1	1	1	1	1	1	2
Haplo_227	2	2	2	2	1	1	1	1	1	1	1	1	1	1	1	1	2	1	2	1	1	2	1	1	2	1	1	1	2	2	1
Haplo_228	2	2	2	2	1	1	1	1	1	1	1	1	2	1	1	1	1	1	1	1	1	2	1	1	2	1	1	2	2	1	1
Haplo_229	2	2	2	2	1	1	1	1	1	1	1	2	1	1	1	1	1	1	1	1	1	2	1	1	1	1	1	1	2	2	1
Haplo_23	1	1	2	2	1	1	1	1	2	1	1	1	2	1	1	1	1	1	1	1	1	1	2	1	1	1	1	1	1	1	2
Haplo_231	1	2	1	1	1	1	1	1	1	1	1	2	1	1	1	1	1	1	1	1	1	2	1	1	1	1	1	1	2	2	1
Haplo_232	1	2	1	1	2	1	2	1	1	1	2	1	1	1	1	1	1	1	1	1	1	1	2	1	1	1	1	1	1	1	2
Haplo_233	1	2	1	2	1	1	1	1	1	1	1	1	1	1	1	1	2	1	2	1	1	2	1	1	2	1	1	1	2	2	1
Haplo_234	1	2	1	2	1	1	1	1	1	1	1	2	1	1	1	1	1	1	1	1	1	2	1	1	1	1	1	1	2	2	1
Haplo_237	1	2	1	2	2	1	2	2	1	1	1	1	2	1	1	1	1	1	1	1	1	1	1	1	2	2	1	1	2	2	1
Haplo_238	1	2	2	1	1	1	1	1	1	1	1	1	1	1	1	1	1	1	1	1	1	1	2	1	1	1	1	1	1	1	2
Haplo_239	1	1	2	2	2	1	1	1	1	1	1	1	1	1	1	1	1	1	1	1	1	1	1	1	1	1	1	1	2	2	1
Haplo_24	1	1	1	1	1	1	1	1	1	1	1	1	1	1	1	2	1	1	1	1	1	1	2	2	1	1	1	1	1	1	2
Haplo_240	1	1	2	2	2	1	1	1	1	1	1	1	1	1	1	1	1	1	1	1	1	1	1	1	1	1	1	1	2	2	1
Haplo_242	1	1	2	1	1	1	1	1	1	1	1	1	1	1	1	1	1	1	1	1	1	1	2	1	1	1	1	1	2	2	1
Haplo_243	1	1	2	1	1	1	1	1	1	1	1	1	1	1	1	2	1	1	1	1	1	1	2	2	1	1	1	1	1	1	2
Haplo_244	1	1	2	2	1	1	1	1	1	1	1	1	1	1	1	1	2	1	2	1	1	2	1	1	2	1	1	1	2	2	1
Haplo_246	1	1	2	2	1	1	1	1	1	1	1	1	2	1	1	1	1	1	1	1	1	1	1	1	2	1	2	1	2	2	1
Haplo_247	1	1	2	2	1	1	1	1	1	1	1	1	2	1	1	1	1	1	1	1	1	2	1	1	2	1	1	2	2	1	1
Haplo_248	1	1	2	2	1	1	1	1	1	1	1	2	1	1	1	1	1	1	1	1	1	2	1	1	1	1	1	1	2	2	1
Haplo_25	1	1	1	2	1	1	1	1	1	1	1	1	1	1	1	1	1	1	1	1	1	1	2	1	1	1	1	1	1	1	2
Haplo_250	2	2	2	2	1	1	1	1	2	2	1	1	2	1	1	1	1	2	1	1	1	1	1	1	2	1	1	1	2	2	1
Haplo_251	1	1	1	1	1	1	1	1	1	1	1	1	1	1	1	1	1	1	1	1	1	1	2	1	1	1	1	1	1	1	2
Haplo_252	2	2	2	1	2	1	2	2	1	1	1	1	2	1	1	1	1	1	1	1	1	1	1	1	2	1	1	1	2	2	1
Haplo_253	2	2	2	2	1	1	1	1	1	1	1	1	2	1	1	1	1	1	1	1	1	1	1	1	2	1	1	1	2	2	1
Haplo_254	2	2	2	2	1	1	1	1	1	1	1	1	1	1	1	1	1	1	1	1	1	2	1	1	1	1	1	1	2	2	2
Haplo_255	1	2	1	2	1	1	1	1	1	1	1	1	2	1	1	1	1	1	1	1	1	1	1	1	2	1	1	2	1	1	2
Haplo_256	2	2	2	2	1	1	1	1	1	1	1	1	1	1	1	1	1	1	1	1	1	2	1	1	1	1	1	1	2	2	2
Haplo_257	1	1	2	2	1	1	1	1	1	1	1	2	1	1	1	1	1	1	1	1	1	1	1	1	2	1	1	2	1	1	2
Haplo_258	1	1	1	2	2	1	2	2	1	1	1	1	2	1	1	1	1	1	1	1	1	1	1	1	2	2	1	1	2	2	1
Haplo_259	1	1	2	1	1	1	1	1	1	1	1	1	2	1	1	1	2	1	1	1	1	1	1	1	2	1	1	1	2	2	1
Haplo_26	1	1	1	2	1	1	1	1	1	1	1	1	2	1	1	1	1	1	1	1	1	1	1	1	2	1	1	2	1	1	2
Haplo_260	1	1	2	2	1	1	1	1	1	1	1	1	2	1	1	1	1	1	1	1	1	1	1	1	2	1	2	1	2	2	1
Haplo_261	1	1	2	2	1	1	1	1	1	1	1	1	2	1	1	1	1	2	1	1	1	1	1	1	2	1	1	1	2	2	1
Haplo_262	1	1	2	1	1	1	1	1	1	1	1	1	1	1	1	1	1	1	1	1	1	1	2	1	1	1	1	1	1	1	2
Haplo_263	1	1	2	2	1	1	1	1	1	1	1	1	2	1	1	1	1	1	1	1	1	1	1	1	2	1	2	1	2	2	1
Haplo_264	1	1	2	2	1	1	1	1	2	2	1	1	2	1	1	1	1	2	1	1	1	1	1	1	2	1	1	1	2	2	1
Haplo_265	1	1	2	2	2	1	1	1	1	1	1	1	1	1	1	1	1	1	1	1	1	1	1	1	1	1	1	1	2	2	1
Haplo_266	1	1	2	2	1	1	1	1	1	1	1	1	2	1	1	1	1	1	1	1	1	1	1	1	2	1	1	2	1	1	2
Haplo_267	1	1	2	1	1	1	1	1	1	1	1	1	1	1	1	1	1	1	1	1	1	1	1	1	1	1	1	1	2	2	1
Haplo_268	1	1	1	2	1	1	1	1	1	1	1	1	2	1	1	1	1	2	1	1	1	1	1	1	2	1	1	1	2	2	1
Haplo_269	1	1	2	2	1	1	1	1	1	1	1	1	1	1	1	1	2	1	1	1	1	2	1	1	2	1	1	1	2	2	1
Haplo_27	1	1	2	1	1	1	1	1	1	1	1	1	1	1	1	1	1	1	1	1	1	1	1	1	1	1	1	1	2	2	1
Haplo_270	1	1	2	2	1	1	1	1	1	1	1	1	2	1	1	1	1	1	1	1	1	1	1	1	2	1	1	2	1	1	2
Haplo_271	1	1	2	2	1	1	1	1	1	1	1	1	2	1	1	1	1	1	1	1	1	2	1	1	2	1	1	1	2	2	1
Haplo_272	1	1	2	2	1	1	1	1	1	1	1	1	2	1	1	1	1	2	1	1	1	1	1	1	2	1	1	1	2	2	1
Haplo_273	1	1	2	2	1	1	1	1	2	2	1	1	2	1	1	1	1	2	1	1	1	1	1	1	2	1	1	1	2	2	1
Haplo_274	1	1	2	2	2	1	1	1	1	1	1	1	1	1	1	1	1	1	1	1	1	1	1	1	1	1	1	1	2	2	2
Haplo_275	1	1	2	2	2	1	2	2	1	1	1	1	2	1	1	1	1	1	1	1	1	1	1	1	2	2	1	1	2	2	1
Haplo_276	1	1	2	2	2	1	2	2	1	1	1	1	2	1	1	1	1	1	1	1	1	2	1	1	2	1	1	2	1	1	2
Haplo_277	1	1	1	1	1	1	1	1	1	1	1	1	1	1	1	1	1	1	1	1	1	1	2	1	1	1	1	1	1	1	2
Haplo_279	1	1	1	2	1	1	1	1	1	1	1	2	1	1	1	1	1	1	1	1	1	2	1	1	1	1	1	1	2	2	1
Haplo_282	1	1	1	1	1	1	1	1	1	1	1	1	1	1	1	1	1	1	1	1	1	1	2	1	1	1	1	1	2	2	1
Haplo_283	1	1	1	2	1	1	1	1	1	1	1	1	1	1	1	1	2	1	2	1	1	2	1	1	2	1	1	1	2	2	1
Haplo_284	1	1	1	2	1	1	1	1	1	1	1	1	2	1	1	1	1	1	1	1	1	1	1	1	2	1	1	2	1	1	2
Haplo_285	1	1	1	2	2	1	2	2	1	1	1	1	2	1	1	1	1	1	1	1	1	1	1	1	2	2	1	1	2	2	1
Haplo_286	1	2	1	1	1	1	1	1	1	1	1	1	1	1	1	1	1	1	1	1	1	1	2	1	1	1	1	1	1	1	2
Haplo_287	1	2	1	2	1	1	1	1	1	1	1	1	1	1	1	1	2	1	2	1	1	2	1	1	2	1	1	1	2	2	1

Haplotype nr	America	East Asia	Europe	Middle East	Central South Asia	Oceania	Subsaharian Africa	rs10265249	rs7458593	rs4723034	rs7384927	rs10225302	rs13438031	rs28371550	rs6954044	rs33995922	rs28371551	rs17159769	rs28371552	rs28371553	rs17159772	rs28371554	rs28371555	rs28371556	rs28371557	rs2302019	rs2302020	rs2302021	rs4988494	rs2267721	rs2267723	rs4988496	rs28371558	rs28371559	rs4988498	rs4988499	rs740336	rs28371560	rs2741	rs4988505	rs28371561	rs28371562	rs2228078	rs2586	rs28371563	rs28371564	rs28371565	rs28371566	rs6962369	rs10653361	rs4723039	rs28371567	rs28371568	rs28371569	rs28371570	rs11771444	rs11973385	rs11760522
Haplo_288	0%	0%	0%	0%	0.25%	0%	0%	2	2	2	2	2	2	2	1	1	1	2	2	1	2	1	1	1	1	2	2	1	1	2	1	1	1	1	1	1	1	1	1	1	1	1	1	1	1	1	1	1	1	2	1	1	1	1	1	1	1	2
Haplo_289	0%	0%	0%	0.31%	0%	0%	0%	2	2	2	2	2	2	2	1	1	1	2	2	1	2	1	1	1	1	2	2	1	1	2	2	1	1	1	1	1	1	1	1	1	1	1	1	1	1	1	1	1	1	2	1	1	1	1	1	1	1	2
Haplo_290	0%	0.43%	0%	0.61%	0%	0%	0%	1	1	1	1	1	1	1	1	1	1	1	1	1	1	1	1	1	1	1	1	1	1	1	1	1	1	1	1	1	1	1	1	1	1	1	1	1	1	1	1	1	1	2	1	1	1	1	1	1	1	2
Haplo_291	0%	0.22%	0%	0.31%	0%	0%	0%	1	1	1	1	2	1	1	1	2	1	1	1	1	1	1	1	1	1	2	1	1	2	1	1	1	1	1	1	1	1	1	1	1	1	1	1	1	1	1	1	1	1	2	1	1	1	1	1	1	1	2
Haplo_293	0.78%	0%	0%	0%	0%	0%	0%	1	1	1	2	1	1	1	1	1	1	1	1	1	1	1	1	1	1	1	1	1	1	1	2	1	1	1	1	1	1	1	1	2	1	1	1	1	1	1	1	1	1	1	1	2	1	1	2	1	1	2
Haplo_294	0%	0.86%	0%	0%	0%	1.79%	0%	1	1	1	1	1	1	1	1	1	1	1	1	1	1	1	1	1	1	1	1	1	1	1	1	1	1	1	1	1	1	1	1	1	1	1	1	1	1	1	1	1	1	2	1	1	1	1	1	1	1	2
Haplo_295	0%	0%	0%	0.31%	0%	0%	0%	1	1	1	2	2	2	2	1	1	1	2	2	1	2	1	1	1	1	2	2	1	1	1	2	2	1	2	2	1	1	1	1	2	1	1	1	1	1	1	1	1	1	1	1	2	2	1	1	2	2	1
Haplo_296	0%	0.65%	0%	0%	0%	0%	0%	1	1	1	2	2	2	2	1	1	1	2	2	1	2	1	1	1	1	2	2	1	1	2	1	1	1	1	1	1	1	1	1	1	1	1	1	1	1	1	1	1	1	2	1	1	1	1	1	1	1	2
Haplo_297	0%	0%	0%	0%	0.25%	0%	0%	1	1	2	1	1	1	1	1	2	1	1	1	1	1	1	1	1	1	2	1	1	2	1	2	1	1	1	1	1	1	1	1	2	1	1	1	1	1	1	1	1	1	1	1	2	1	1	2	1	1	2
Haplo_298	0%	0%	0%	0%	0.25%	0%	0%	1	1	2	1	2	1	1	1	2	1	1	1	1	1	1	1	1	1	2	1	1	2	1	2	1	1	1	1	2	2	1	1	2	1	1	1	1	2	1	1	1	1	1	1	2	1	1	1	2	2	1
Haplo_299	0%	0.22%	0%	0%	0%	0%	0%	1	1	2	2	2	1	1	1	1	1	1	2	1	1	1	1	1	1	2	1	2	2	2	1	1	1	1	1	1	1	1	1	1	1	1	1	1	1	1	1	1	1	2	1	1	1	1	1	1	1	2
Haplo_30	0%	0%	0%	0%	0%	0%	0.48%	1	1	1	2	2	2	2	1	1	1	2	2	1	2	1	1	1	1	2	2	1	1	2	1	1	1	1	1	1	1	1	1	1	1	1	1	1	1	1	2	1	1	2	1	1	1	1	1	2	2	1
Haplo_300	0%	0%	0%	0%	0%	0%	0.48%	1	1	2	2	2	2	2	1	1	1	2	2	1	2	1	1	1	1	2	2	1	1	1	2	2	1	2	2	1	1	1	1	2	1	1	1	1	1	1	1	1	1	1	1	2	2	1	1	2	2	1
Haplo_301	0%	0%	0%	0%	0%	0%	0.48%	1	1	2	2	2	2	2	1	1	1	2	2	1	2	1	1	1	1	2	2	1	1	2	2	2	1	2	2	1	1	1	1	2	1	1	1	1	1	1	1	1	1	1	1	2	2	1	1	2	2	1
Haplo_304	0%	0%	0%	0%	0%	3.57%	0%	1	1	1	1	1	1	1	1	1	1	1	1	1	1	1	1	1	1	1	1	1	1	1	1	1	1	1	1	1	1	1	1	1	1	1	1	1	1	1	1	1	1	2	1	1	1	1	2	1	1	2
Haplo_305	0%	0%	0%	1.23%	0%	0%	0.48%	1	1	1	1	1	1	1	1	1	1	1	1	1	1	1	1	1	1	1	1	1	1	1	1	1	1	1	1	1	1	1	1	1	1	1	2	1	1	1	1	1	1	2	1	1	1	1	1	1	1	2
Haplo_307	0%	0%	0%	0.31%	0%	0%	0%	1	1	1	1	1	1	1	1	1	1	1	1	1	1	1	1	1	1	1	1	1	1	1	1	1	1	1	1	1	1	1	1	2	1	1	1	1	1	1	1	1	1	1	1	2	2	1	1	2	2	1
Haplo_308	0%	0%	0%	0.61%	0%	0%	0%	1	1	1	1	1	1	1	1	1	1	1	1	1	1	1	1	1	1	1	1	1	1	1	1	1	1	1	1	1	1	1	2	1	1	1	1	1	1	1	1	1	1	2	1	1	1	1	1	1	1	2
Haplo_309	0%	0%	0%	0.92%	0%	0%	0%	1	1	1	1	1	1	1	1	1	1	1	1	1	1	1	1	1	1	1	1	1	1	1	1	1	1	1	1	1	1	1	2	1	1	1	1	1	1	1	1	1	2	1	1	1	1	1	1	2	2	1
Haplo_31	0%	0%	0%	0%	0.25%	0%	0%	1	1	1	2	2	2	2	1	1	1	2	2	1	2	1	1	1	1	2	2	1	1	2	1	1	1	1	1	1	1	1	1	2	1	1	1	1	1	1	1	1	1	1	1	1	2	1	1	1	1	2
Haplo_310	0%	0.22%	0%	0%	0%	0%	0%	1	1	1	1	1	1	1	1	1	1	1	1	1	1	1	1	1	1	1	1	1	1	1	1	1	1	1	1	1	1	2	1	1	1	1	1	1	1	1	1	1	1	2	1	1	1	1	1	1	1	2
Haplo_311	0%	0.86%	0%	0%	0.50%	0%	0%	1	1	1	1	1	1	1	1	1	1	1	1	1	1	1	1	1	1	1	1	1	1	1	1	2	1	2	1	1	1	2	1	1	1	1	1	1	1	1	1	1	1	2	1	1	1	1	1	1	1	2
Haplo_312	0%	0%	0%	0%	0.25%	0%	0%	1	1	1	1	1	1	1	1	1	1	1	1	1	1	1	1	1	1	1	1	1	1	1	1	2	1	2	1	1	1	2	1	2	1	1	1	1	1	1	1	1	1	1	1	2	1	1	2	1	1	2
Haplo_313	0%	0%	0%	0.31%	0%	0%	0%	1	1	1	1	1	1	1	1	1	1	1	1	1	1	1	1	1	1	1	1	1	1	1	2	1	1	1	1	1	1	1	1	1	1	1	1	1	1	1	1	1	1	1	1	2	1	1	1	2	2	1
Haplo_318	0%	0%	0%	0%	0.25%	0%	0%	1	1	1	1	1	1	1	1	1	1	1	1	1	1	1	1	1	1	1	1	1	1	1	2	1	1	1	1	1	1	1	1	2	1	1	1	1	1	1	1	1	2	1	1	2	1	1	2	2	1	1
Haplo_319	0%	0%	0%	0%	0.25%	0%	0%	1	1	1	1	1	1	1	1	1	1	1	1	1	1	1	1	1	1	1	1	1	1	1	2	1	1	1	1	1	1	1	1	2	1	1	1	1	1	1	1	1	2	1	2	2	1	1	2	2	1	1
Haplo_320	2.34%	0.65%	0%	0%	0.25%	0%	0%	1	1	1	1	1	1	1	1	1	1	1	1	1	1	1	1	1	1	1	1	1	1	1	2	1	1	1	1	1	1	1	2	1	1	1	1	1	1	1	1	1	2	1	1	1	1	1	1	1	1	2
Haplo_322	0%	0%	0%	0%	0.25%	0%	0%	1	1	1	1	1	1	1	1	1	1	1	1	1	1	1	1	1	1	1	1	1	1	1	2	1	1	1	1	1	1	1	2	1	1	1	1	1	1	1	1	1	2	1	1	1	1	1	2	1	1	2
Haplo_323	0%	0%	0%	0.31%	0.74%	5.36%	0%	1	1	1	1	1	1	1	1	1	1	1	1	1	1	1	1	1	1	1	1	1	1	1	2	1	1	1	1	2	2	1	1	2	1	1	1	1	2	1	1	1	1	1	1	2	1	1	1	2	2	1
Haplo_327	0%	0%	0%	0.31%	0%	0%	0%	1	1	1	1	1	1	1	1	1	1	1	1	1	1	1	1	1	1	1	1	1	1	2	1	1	1	1	1	1	1	1	1	1	1	1	2	1	1	1	1	1	1	2	1	1	1	1	1	1	1	2
Haplo_329	0%	0%	0%	0%	0.25%	0%	0%	1	1	1	1	1	1	1	1	1	1	1	1	1	1	1	1	1	1	2	1	1	2	2	1	1	1	1	1	1	1	1	1	1	1	1	1	1	1	1	1	1	1	2	1	1	1	1	1	1	1	2
Haplo_33	0%	0%	0%	0.92%	0%	0%	0%	1	1	1	2	2	2	2	1	1	1	2	2	1	2	1	1	1	1	2	2	1	1	2	1	1	1	1	1	1	1	1	2	1	1	1	1	1	1	1	1	1	2	1	1	1	1	1	1	2	2	1
Haplo_330	0%	0.22%	0%	0%	0%	0%	0%	1	1	1	1	1	1	1	1	1	1	1	1	1	1	1	1	1	1	2	2	1	1	2	1	1	1	1	1	1	1	1	1	1	1	1	1	1	1	1	1	1	1	2	1	1	1	1	1	1	1	2
Haplo_331	0%	0.43%	0%	0%	0%	0%	0%	1	1	1	1	1	1	1	1	1	1	1	1	1	1	1	1	1	1	2	2	1	1	2	2	1	1	1	1	1	1	1	2	1	1	1	1	1	1	1	1	1	2	1	1	1	1	1	1	2	2	1
Haplo_332	0.78%	0%	0%	0%	0%	0%	0%	1	1	1	1	1	1	1	1	1	1	1	1	1	1	2	1	1	1	1	1	1	1	1	1	1	1	1	1	1	1	1	1	1	1	1	1	1	1	1	1	1	1	2	1	1	1	1	1	1	1	2
Haplo_333	1.56%	0%	0%	0%	0%	0%	0%	1	1	1	1	1	1	1	1	1	1	1	1	1	1	2	1	1	1	1	1	1	1	1	1	1	1	1	1	1	1	1	1	1	1	1	2	1	1	1	1	1	1	2	2	1	1	1	1	1	1	2
Haplo_334	0.78%	0%	0%	0%	0%	0%	0%	1	1	1	1	1	1	1	1	1	1	1	1	1	1	2	1	1	1	1	1	1	1	1	2	1	1	1	1	1	1	1	1	2	1	1	1	1	1	1	1	1	1	1	1	2	1	1	2	1	1	2
Haplo_335	0%	0.22%	0%	0%	0%	0%	0%	1	1	1	1	1	1	1	1	1	1	1	2	1	1	1	1	1	1	2	1	2	2	1	1	1	1	1	1	1	1	1	1	1	1	1	1	1	1	1	1	1	1	2	1	1	1	1	1	1	1	2
Haplo_336	0%	0%	0%	0%	0.25%	0%	0%	1	1	1	1	1	1	1	1	1	1	1	2	1	1	1	1	1	1	2	1	2	2	1	1	2	1	2	1	1	1	2	1	1	1	1	1	1	1	1	1	1	1	2	1	1	1	1	1	1	1	2
Haplo_337	0%	0.65%	0%	0%	0%	0%	0%	1	1	1	1	1	1	1	1	1	1	1	2	1	1	1	1	1	1	2	1	2	2	2	1	1	1	1	1	1	1	1	1	1	1	1	1	1	1	1	1	1	1	2	1	1	1	1	1	1	1	2
Haplo_338	0%	1.51%	0%	0%	0.25%	0%	0%	1	1	1	1	1	1	1	1	1	1	1	2	1	1	1	1	1	1	2	1	2	2	2	2	1	1	1	1	1	1	1	2	1	1	1	1	1	1	1	1	1	2	1	1	1	1	1	1	1	1	2
Haplo_339	0%	0.43%	0%	0%	0.50%	0%	0%	1	1	1	1	1	1	1	1	1	1	1	2	1	1	1	1	1	1	2	1	2	2	2	2	1	1	1	1	1	1	1	2	1	1	1	1	1	1	1	1	1	2	1	1	1	1	1	1	2	2	1
Haplo_34	0%	0.22%	0%	0%	0%	0%	0%	1	1	1	2	2	2	2	1	1	1	2	2	1	2	1	1	1	1	2	2	1	1	2	1	1	1	1	1	2	2	1	1	2	1	1	1	1	2	1	1	1	1	1	1	2	1	1	1	2	2	1
Haplo_340	0%	0.22%	0%	0%	0%	0%	0%	1	1	1	1	1	1	1	1	2	1	1	1	1	1	1	1	1	1	2	1	1	2	1	1	2	1	2	1	1	1	2	1	1	1	1	1	1	1	1	1	1	1	2	1	1	1	1	1	1	1	2
Haplo_341	0%	0%	0%	0.31%	0%	0%	0%	1	1	1	1	1	1	1	1	2	1	1	1	1	1	1	1	1	1	2	1	1	2	1	2	1	1	1	1	1	1	1	1	1	1	1	1	2	1	2	1	1	2	1	1	2	1	1	1	2	2	1
Haplo_342	0%	0%	0%	0%	0.25%	0%	0%	1	1	1	1	1	1	1	1	2	1	1	1	1	1	1	1	1	1	2	1	1	2	1	2	1	1	1	1	1	1	1	1	2	1	1	1	1	1	1	1	1	1	1	1	2	1	1	2	1	1	2
Haplo_343	0%	0%	0%	0.61%	0%	0%	0%	1	1	1	1	1	1	1	1	2	1	1	1	1	1	1	1	1	1	2	1	1	2	1	2	1	1	1	1	1	1	1	2	1	1	1	1	1	1	1	1	1	2	1	1	1	1	1	1	2	2	1
Haplo_344	0%	0.22%	0%	0%	0%	0%	0%	1	1	1	1	1	1	1	1	2	1	1	1	1	1	1	1	1	1	2	2	1	1	2	2	1	1	1	1	1	1	1	2	1	1	1	1	1	1	1	1	1	2	1	1	1	1	1	1	2	2	1
Haplo_346	0%	0%	0%	0.92%	0%	0%	0%	1	1	1	1	1	1	1	2	1	1	1	1	1	1	1	1	1	1	1	1	1	1	1	1	1	1	1	1	1	1	1	1	1	1	1	1	1	1	1	1	1	1	2	1	1	1	1	1	2	2	1
Haplo_347	0%	0%	0%	0.31%	0%	0%	0%	1	1	1	1	1	1	1	2	1	1	1	1	1	1	1	1	1	1	1	1	1	1	1	2	1	1	1	1	1	1	1	1	1	1	1	1	2	1	2	1	1	2	1	1	2	1	1	1	2	2	1
Haplo_350	0%	0%	0%	0%	0.25%	0%	0%	1	1	1	1	1	1	1	2	1	1	1	1	1	1	1	1	1	1	1	1	1	1	1	2	1	1	1	1	1	1	1	2	1	1	1	1	1	1	1	1	1	2	1	1	1	1	1	1	1	1	2
Haplo_351	0%	0%	0%	0%	0%	1.79%	0%	1	1	1	1	1	2	2	1	1	1	2	2	1	1	1	1	1	1	2	2	1	1	2	2	1	1	1	1	1	1	1	1	2	1	1	1	1	1	1	1	1	2	1	1	2	1	1	2	2	1	1
Haplo_352	0%	0%	0%	0%	0.50%	0%	0%	1	1	1	1	1	2	2	1	1	1	2	2	1	2	1	1	2	1	2	2	1	1	2	2	1	1	1	1	1	1	1	1	2	1	1	1	1	1	1	1	1	1	1	1	2	1	1	2	1	1	2
Haplo_353	0%	0%	0%	0%	0.25%	0%	0%	1	1	1	1	1	2	2	1	1	1	2	2	1	2	1	1	2	1	2	2	1	1	2	2	1	1	1	1	1	1	1	2	1	1	1	1	1	1	1	1	1	1	2	1	1	1	1	1	1	1	2
Haplo_354	0%	1.08%	0%	0.31%	0.99%	0%	0%	1	1	1	1	2	1	1	1	1	1	1	1	1	1	1	1	1	1	1	1	1	1	1	1	1	1	1	1	1	1	1	1	1	1	1	1	1	1	1	1	1	1	2	1	1	1	1	1	1	1	2
Haplo_355	0%	0%	0%	0%	0.50%	0%	0%	1	1	1	1	2	1	1	1	1	1	1	1	1	1	1	1	1	1	1	1	1	1	1	1	1	1	1	1	1	1	1	1	1	1	1	2	1	1	1	1	1	1	2	2	1	1	1	1	1	1	2
Haplo_356	0%	0%	0%	0%	0.25%	0%	0%	1	1	1	1	2	1	1	1	1	1	1	1	1	1	1	1	1	1	1	1	1	1	1	2	1	1	1	1	1	1	1	1	1	1	1	1	1	1	1	1	1	1	2	1	1	1	1	1	1	1	2
Haplo_357	0%	0%	0%	0.31%	0%	0%	0%	1	1	1	1	2	1	1	1	1	1	1	1	1	1	1	1	1	1	1	1	1	1	1	2	1	1	1	1	1	1	1	1	2	1	1	1	1	1	1	1	1	1	1	1	2	1	1	2	1	1	2
Haplo_358	0%	0%	0%	0%	0.25%	1.79%	0%	1	1	1	1	2	1	1	1	1	1	1	1	1	1	1	1	1	1	1	1	1	1	1	2	1	1	1	1	1	1	1	2	1	1	1	1	1	1	1	1	1	2	1	1	1	1	1	1	2	2	1
Haplo_359	0%	0.22%	0%	0%	0%	0%	0%	1	1	1	1	2	1	1	1	1	1	1	1	1	1	1	1	1	1	1	1	1	1	1	2	1	1	1	1	2	2	1	1	2	1	1	1	1	2	1	1	1	1	1	1	2	1	1	1	2	2	1
Haplo_360	0%	0%	0%	0%	0%	0%	0.95%	1	1	1	1	2	1	1	1	1	1	1	1	1	1	1	1	1	1	2	1	1	2	1	2	1	1	1	1	1	1	1	1	1	1	1	1	1	1	1	1	1	2	1	1	1	1	1	1	2	2	1
Haplo_361	0%	0%	0%	0.31%	0%	0%	0%	1	1	1	1	2	1	1	1	1	1	1	1	1	1	1	1	1	1	2	1	1	2	1	2	1	1	1	1	1	1	1	2	1	1	1	1	1	1	1	1	1	2	1	1	1	1	1	1	2	2	1
Haplo_363	0%	0%	0%	0%	0.50%	0%	0%	1	1	1	1	2	1	1	1	1	1	1	1	1	1	1	1	1	1	2	1	1	2	2	2	1	1	1	1	2	2	1	1	2	1	1	1	1	2	1	1	1	1	1	1	2	1	1	1	2	2	1
Haplo_364	0%	0%	0%	0%	0.25%	0%	0%	1	1	1	1	2	1	1	1	1	1	1	1	1	1	1	1	1	1	2	1	2	2	2	2	1	1	1	1	1	1	1	2	1	1	1	1	1	1	1	1	1	2	1	1	1	1	1	1	2	2	1
Haplo_366	0%	0%	0%	0%	0.25%	0%	0%	1	1	1	1	2	1	1	1	1	1	1	2	1	1	1	1	1	1	2	1	2	2	2	1	1	1	1	1	1	1	1	1	1	1	1	2	1	1	1	1	1	1	2	2	1	1	1	1	1	1	2
Haplo_367	0%	0.22%	0%	0%	0%	0%	0%	1	1	1	1	2	1	1	1	1	1	1	2	1	1	1	1	1	1	2	1	2	2	2	2	1	1	1	1	1	1	1	1	1	1	1	1	1	1	1	1	1	1	2	1	1	1	1	1	1	1	2

Haplotype nr	America	East Asia	Europe	Middle East	Central South Asia	Oceania	Subsaharian Africa	rs10265249	rs7458593	rs4723034	rs7384927	rs10225302	rs13438031	rs28371550	rs6954044	rs33995922	rs28371551	rs17159769	rs28371552	rs28371553	rs17159772	rs28371554	rs28371555	rs28371556	rs28371557	rs2302019	rs2302020	rs2302021	rs4988494	rs2267721	rs2267723	rs4988496	rs28371558	rs28371559	rs4988498	rs4988499	rs740336	rs28371560	rs2741	rs4988505	rs28371561	rs28371562	rs2228078	rs2586	rs28371563	rs28371564	rs28371565	rs28371566	rs6962369	rs10653361	rs4723039	rs28371567	rs28371568	rs28371569	rs28371570	rs11771444	rs11973385	rs11760522
Haplo_368	0%	0%	0%	0.92%	0%	0%	0%	1	1	1	1	2	1	1	1	1	1	1	2	1	1	1	1	1	1	2	1	2	2	2	2	1	1	1	1	1	1	1	1	1	1	1	1	2	1	2	1	1	2	1	1	2	1	1	1	2	2	1
Haplo_369	0%	0%	0%	0.31%	0%	0%	0%	1	1	1	1	2	1	1	1	1	1	1	2	1	1	1	1	1	1	2	1	2	2	2	2	1	1	1	1	1	1	1	1	2	1	1	1	1	1	1	1	1	1	1	1	2	1	1	2	1	1	2
Haplo_37	0%	0%	0%	0.92%	0%	1.79%	0%	1	1	1	2	2	2	2	1	1	1	2	2	1	2	1	1	1	1	2	2	1	1	2	2	1	1	1	1	1	1	1	1	1	1	1	1	1	1	1	1	1	1	2	1	1	1	1	1	1	1	2
Haplo_370	0.78%	0%	0%	0%	0%	0%	0%	1	1	1	1	2	1	1	1	1	1	1	2	1	1	1	1	1	1	2	1	2	2	2	2	1	1	1	1	1	1	1	2	1	1	1	1	1	1	1	1	1	2	1	1	1	1	1	1	1	1	2
Haplo_371	0%	2.37%	0%	1.23%	0.50%	0%	0%	1	1	1	1	2	1	1	1	1	1	1	2	1	1	1	1	1	1	2	1	2	2	2	2	1	1	1	1	1	1	1	2	1	1	1	1	1	1	1	1	1	2	1	1	1	1	1	1	2	2	1
Haplo_372	0%	0%	0%	0.31%	0%	0%	0%	1	1	1	1	2	1	1	1	1	1	1	2	1	1	1	1	1	1	2	1	2	2	2	2	1	1	1	1	2	2	1	1	2	1	1	1	1	2	1	1	1	1	1	1	2	1	1	1	2	2	1
Haplo_373	0%	0%	0%	0%	0.25%	0%	0%	1	1	1	1	2	1	1	1	1	1	1	2	1	1	1	1	1	1	2	1	2	2	2	2	2	1	1	1	1	1	1	1	1	1	1	1	1	1	1	1	1	1	1	1	1	1	1	1	2	2	1
Haplo_374	0%	0%	0%	0%	0.25%	0%	0%	1	1	1	1	2	1	1	1	1	1	1	2	1	1	1	1	1	1	2	1	2	2	2	2	2	1	2	2	1	1	1	1	2	1	1	1	1	2	1	1	1	1	1	1	2	1	1	1	2	2	1
Haplo_376	0%	0%	0%	1.23%	0%	0%	0%	1	1	1	1	2	1	1	1	2	1	1	1	1	1	1	1	1	1	2	1	1	2	1	1	1	1	1	1	1	1	1	1	1	1	1	2	1	1	1	1	1	1	2	2	1	1	1	1	1	1	2
Haplo_377	0%	0.86%	0%	0%	0%	0%	0%	1	1	1	1	2	1	1	1	2	1	1	1	1	1	1	1	1	1	2	1	1	2	1	1	2	1	2	1	1	1	2	1	1	1	1	1	1	1	1	1	1	1	2	1	1	1	1	1	1	1	2
Haplo_378	0%	0%	0%	1.23%	0.74%	0%	0%	1	1	1	1	2	1	1	1	2	1	1	1	1	1	1	1	1	1	2	1	1	2	1	2	1	1	1	1	1	1	1	1	1	1	1	1	1	1	1	1	1	1	2	1	1	1	1	1	1	1	2
Haplo_38	0%	0%	0%	0%	0.50%	0%	0%	1	1	1	2	2	2	2	1	1	1	2	2	1	2	1	1	1	1	2	2	1	1	2	2	1	1	1	1	1	1	1	1	1	1	1	1	1	1	1	1	1	2	1	1	1	1	1	1	2	2	2
Haplo_381	0%	0%	0%	0.31%	0%	0%	0%	1	1	1	1	2	1	1	1	2	1	1	1	1	1	1	1	1	1	2	1	1	2	1	2	1	1	1	1	1	1	1	1	2	1	1	1	1	1	1	1	1	1	1	1	2	2	1	1	2	2	1
Haplo_384	0%	0%	0%	0.31%	0%	0%	0%	1	1	1	1	2	1	1	1	2	1	1	1	1	1	1	1	1	1	2	1	1	2	1	2	2	1	1	1	1	1	1	1	1	1	1	1	2	1	1	1	2	2	1	1	2	1	1	1	2	2	2
Haplo_385	0%	0%	0%	0.31%	0%	0%	0%	1	1	1	1	2	2	1	1	1	1	2	2	1	1	1	1	1	1	2	2	1	1	2	2	1	1	1	1	1	1	1	1	1	1	1	1	1	1	1	1	1	1	2	1	1	1	1	1	1	1	2
Haplo_386	0%	0.22%	0%	0%	0%	0%	0%	1	1	1	1	2	2	2	1	1	1	1	2	1	2	1	1	1	1	2	1	1	1	2	1	1	1	1	1	1	1	1	1	1	1	1	1	1	1	1	1	1	1	2	1	1	1	1	1	1	1	2
Haplo_39	0%	0%	0%	0.31%	0%	0%	0%	1	1	1	2	2	2	2	1	1	1	2	2	1	2	1	1	1	1	2	2	1	1	2	2	1	1	1	1	1	1	1	1	1	1	1	1	2	1	1	1	2	2	1	1	2	1	1	1	2	2	2
Haplo_390	0%	0%	0%	0%	0.25%	0%	0%	1	1	1	1	2	2	2	1	1	1	2	2	1	2	1	1	1	1	2	2	1	1	2	1	1	1	1	1	1	1	1	1	1	1	1	1	2	1	2	1	1	2	1	1	2	1	1	1	2	2	1
Haplo_391	0%	0.22%	0%	0%	0%	0%	0%	1	1	1	1	2	2	2	1	1	1	2	2	1	2	1	1	1	1	2	2	1	1	2	2	1	1	1	1	1	1	1	1	1	1	1	1	1	1	1	1	1	1	2	1	1	1	1	1	1	1	2
Haplo_392	0%	0%	0%	0%	0.50%	0%	0%	1	1	1	1	2	2	2	1	1	1	2	2	1	2	1	1	1	1	2	2	1	1	2	2	1	1	1	1	1	1	1	1	1	1	1	1	1	1	1	1	1	2	1	1	1	1	1	1	2	2	1
Haplo_393	0%	0%	0%	0.31%	0%	0%	0%	1	1	1	1	2	2	2	1	1	1	2	2	1	2	1	1	1	1	2	2	1	1	2	2	1	1	1	1	1	1	1	1	1	1	1	1	2	1	1	1	2	2	1	1	2	1	1	1	2	2	2
Haplo_394	0%	0%	0%	0.31%	0.74%	0%	0%	1	1	1	1	2	2	2	1	1	1	2	2	1	2	1	1	1	1	2	2	1	1	2	2	1	1	1	1	1	1	1	1	1	1	1	1	2	1	2	1	1	2	1	1	2	1	1	1	2	2	1
Haplo_396	0%	0.22%	0%	0%	0%	0%	0%	1	1	1	1	2	2	2	1	1	1	2	2	1	2	1	1	1	1	2	2	1	1	2	2	1	1	1	1	1	1	1	2	1	1	1	1	1	1	1	1	1	2	1	1	1	1	1	1	1	1	2
Haplo_399	0%	0%	0%	0%	0.25%	0%	0%	1	1	1	1	2	2	2	1	1	1	2	2	1	2	1	1	1	1	2	2	1	1	2	2	2	1	2	2	1	1	1	1	2	1	1	1	1	1	1	1	1	1	1	1	2	1	1	1	2	2	1
Haplo_4	0%	0%	0%	0%	0.50%	0%	0%	1	1	1	1	2	2	2	1	1	1	2	2	1	2	1	1	1	1	2	2	1	1	2	1	1	1	1	1	1	1	1	1	1	1	1	1	1	1	1	1	1	1	2	1	1	1	1	1	1	1	2
Haplo_40	0%	0%	0%	0.31%	0.25%	0%	0%	1	1	1	2	2	2	2	1	1	1	2	2	1	2	1	1	1	1	2	2	1	1	2	2	1	1	1	1	1	1	1	1	1	1	1	1	2	1	2	1	1	2	1	1	2	1	1	1	2	2	1
Haplo_401	0%	0%	0%	0%	0.25%	0%	0%	1	1	1	1	2	2	2	1	1	1	2	2	1	2	1	1	2	1	2	2	1	1	2	1	1	1	1	1	1	1	1	1	2	1	1	1	1	1	1	1	1	1	1	1	2	1	1	2	1	1	2
Haplo_402	0%	0%	0%	0%	0.25%	0%	0%	1	1	1	1	2	2	2	1	1	1	2	2	1	2	1	1	2	1	2	2	1	1	2	2	1	1	1	1	1	1	1	1	1	1	1	1	2	1	2	1	1	2	1	1	2	1	1	1	2	2	1
Haplo_405	0%	0%	0%	0%	0.25%	0%	0%	1	1	1	1	2	2	2	1	1	1	2	2	1	2	1	1	2	1	2	2	1	1	2	2	1	1	1	1	1	1	1	2	1	1	1	1	1	1	1	1	1	2	1	1	1	1	1	1	2	2	1
Haplo_406	0%	0.43%	0%	0%	0.50%	0%	0%	1	1	1	2	1	1	1	1	1	1	1	1	1	1	1	1	1	1	1	1	1	1	1	1	1	1	1	1	1	1	1	1	1	1	1	1	1	1	1	1	1	1	2	1	1	1	1	1	1	1	2
Haplo_407	0%	0.22%	0%	0%	0%	0%	0%	1	1	1	2	1	1	1	1	1	1	1	1	1	1	1	1	1	1	1	1	1	1	1	1	1	1	1	1	1	1	1	1	1	1	1	1	1	1	1	1	1	2	1	1	1	1	1	1	2	2	2
Haplo_408	0%	0%	0%	0%	0.50%	0%	0%	1	1	1	2	1	1	1	1	1	1	1	1	1	1	1	1	1	1	1	1	1	1	1	1	1	1	1	1	1	1	1	1	1	1	1	2	1	1	1	1	1	1	2	2	1	1	1	1	1	1	2
Haplo_409	0%	0.22%	0%	0%	0%	0%	0%	1	1	1	2	1	1	1	1	1	1	1	1	1	1	1	1	1	1	1	1	1	1	1	1	1	1	1	1	1	1	1	2	1	1	1	1	1	1	1	1	1	2	1	1	1	1	1	1	2	2	1
Haplo_41	0%	0%	0%	0.61%	0%	0%	0%	1	1	1	2	2	2	2	1	1	1	2	2	1	2	1	1	1	1	2	2	1	1	2	2	1	1	1	1	1	1	1	1	2	1	1	1	1	1	1	1	1	1	1	1	2	1	1	1	2	2	1
Haplo_410	0.78%	0%	0%	0%	0%	0%	0%	1	1	1	2	1	1	1	1	1	1	1	1	1	1	1	1	1	1	1	1	1	1	1	2	1	1	1	1	1	1	1	1	2	1	1	1	1	1	1	1	1	1	1	1	2	1	1	2	1	1	2
Haplo_411	0%	0.22%	0%	0%	0%	0%	0%	1	1	1	2	1	1	1	1	1	1	1	1	1	1	1	1	1	1	1	1	1	1	1	2	1	1	1	1	1	1	1	1	2	1	1	1	1	1	1	1	1	2	1	1	2	1	1	2	2	1	1
Haplo_412	0%	0.65%	0%	0%	0%	0%	0%	1	1	1	2	1	1	1	1	1	1	1	1	1	1	1	1	1	1	1	1	1	1	1	2	1	1	1	1	1	1	1	2	1	1	1	1	1	1	1	1	1	2	1	1	1	1	1	1	1	1	2
Haplo_413	0%	0%	0%	0.31%	0%	0%	0%	1	1	1	2	1	1	1	1	1	1	1	1	1	1	1	1	1	1	1	1	1	1	1	2	2	1	2	2	1	1	1	1	2	1	1	1	1	1	1	1	1	1	1	1	2	2	1	1	2	2	1
Haplo_416	0%	0%	0%	0%	0.50%	0%	0%	1	1	1	2	2	1	1	1	2	1	1	1	1	1	1	1	1	1	2	1	1	2	1	2	1	1	1	1	1	1	1	1	1	1	1	1	2	1	2	1	1	2	1	1	2	1	1	1	2	2	1
Haplo_417	0%	0%	0%	0.31%	0%	0%	0%	1	1	1	2	2	2	1	1	1	2	2	2	2	1	1	1	1	1	2	1	1	1	2	2	2	1	1	1	1	1	1	1	1	1	1	1	1	1	1	1	1	1	1	1	1	1	1	1	2	2	1
Haplo_420	0%	0.22%	0%	0.31%	0%	0%	0%	1	1	2	2	2	1	1	1	1	1	1	2	1	1	1	1	1	1	2	1	2	2	2	1	1	1	1	1	1	1	1	1	1	1	1	1	1	1	1	1	1	1	2	1	1	1	1	1	1	1	2
Haplo_421	0%	0.22%	0%	0%	0%	0%	0%	1	1	2	2	2	1	1	1	1	1	1	2	1	1	1	1	1	1	2	1	2	2	2	2	1	1	1	1	1	1	1	1	1	1	1	1	1	1	1	1	1	1	2	1	1	1	1	1	1	1	2
Haplo_422	0%	0.22%	0%	0%	0%	0%	0%	1	1	2	2	2	1	1	1	1	1	1	2	1	1	1	1	1	1	2	1	2	2	2	2	1	1	1	1	1	1	1	2	1	1	1	1	1	1	1	1	1	2	1	1	1	1	1	1	1	1	2
Haplo_423	0%	0.65%	0%	0%	0%	0%	0%	1	1	2	2	2	1	1	1	1	1	1	2	1	1	1	1	1	1	2	1	2	2	2	2	1	1	1	1	1	1	1	2	1	1	1	1	1	1	1	1	1	2	1	1	1	1	1	1	2	2	1
Haplo_43	0%	0%	0%	0%	0%	0%	0.48%	1	1	1	2	2	2	2	1	1	1	2	2	1	2	1	1	1	1	2	2	1	1	2	2	1	1	1	1	1	1	1	1	2	1	1	1	1	1	1	1	1	1	1	1	2	1	2	1	2	2	1
Haplo_44	0%	0%	0%	0%	0%	5.36%	0%	1	1	1	2	2	2	2	1	1	1	2	2	1	2	1	1	1	1	2	2	1	1	2	2	1	1	1	1	1	1	1	1	2	1	1	1	1	1	1	1	1	2	1	1	2	1	1	2	2	1	1
Haplo_45	0%	0%	0%	0.31%	0%	0%	0%	1	1	1	2	2	2	2	1	1	1	2	2	1	2	1	1	1	1	2	2	1	1	2	2	1	1	1	1	1	1	1	1	2	1	1	1	1	2	1	1	1	1	1	1	2	1	1	1	2	2	1
Haplo_46	0%	0%	0%	0%	0.25%	0%	0%	1	1	1	2	2	2	2	1	1	1	2	2	1	2	1	1	1	1	2	2	1	1	2	2	1	1	1	1	1	1	1	2	1	1	1	1	1	1	1	1	1	1	2	1	1	1	1	1	1	1	2
Haplo_48	0%	0%	0%	0.61%	0.50%	0%	2.86%	1	1	1	2	2	2	2	1	1	1	2	2	1	2	1	1	1	1	2	2	1	1	2	2	1	1	1	1	2	2	1	1	2	1	1	1	1	2	1	1	1	1	1	1	2	1	1	1	2	2	1
Haplo_49	0%	0%	0%	0.31%	0%	0%	0%	1	1	1	2	2	2	2	1	1	1	2	2	1	2	1	1	1	1	2	2	1	1	2	2	2	1	1	1	1	1	1	1	1	1	1	1	1	1	1	1	1	1	1	1	1	1	1	1	2	2	1
Haplo_51	0%	0%	0%	0%	0%	0%	0.48%	1	1	1	2	2	2	2	1	1	1	2	2	1	2	1	1	1	1	2	2	1	1	2	2	2	2	2	2	1	1	1	1	1	1	1	1	1	1	1	1	1	2	1	1	2	1	1	1	2	2	1
Haplo_52	0%	0%	0%	0.31%	0%	0%	0%	1	1	1	2	2	2	2	1	1	1	2	2	1	2	1	1	2	1	2	2	1	1	2	2	1	1	1	1	1	1	1	1	2	1	1	1	1	1	1	1	1	1	1	1	2	1	1	2	1	1	2
Haplo_53	0%	0%	0%	0%	0%	0%	0.48%	1	1	2	1	2	1	1	1	1	1	1	1	1	1	1	1	1	1	2	1	1	2	1	1	1	1	1	1	1	1	1	1	1	1	1	1	1	1	1	1	1	1	2	1	1	1	1	1	2	2	1
Haplo_54	0%	0%	0%	0%	0.50%	0%	0%	1	1	2	1	2	1	1	1	2	1	1	1	1	1	1	1	1	1	2	1	1	2	1	1	1	1	1	1	1	1	1	1	1	1	1	1	1	1	1	1	1	1	2	1	1	1	1	1	1	1	2
Haplo_55	0%	0%	0%	0%	0.25%	0%	0%	1	1	2	2	1	1	1	1	2	1	1	1	1	1	1	1	1	1	2	1	1	2	1	1	1	1	1	1	1	1	1	1	1	1	1	1	1	1	1	1	1	1	2	1	1	1	1	1	1	1	2
Haplo_56	0%	0%	0%	0%	0%	0%	0.48%	1	1	2	2	2	1	1	1	1	1	1	1	1	1	1	1	1	1	2	1	1	2	1	1	1	1	1	1	1	1	1	1	2	1	1	1	1	1	1	1	1	1	1	1	2	1	1	1	2	2	1
Haplo_57	0%	0%	0%	0%	0%	0%	1.43%	1	1	2	2	2	1	1	1	1	1	1	1	1	1	1	1	1	1	2	1	1	2	1	2	1	1	1	1	1	1	1	1	2	1	1	1	1	1	1	1	1	1	1	1	2	1	1	1	2	2	1
Haplo_58	0%	0%	0%	0%	0%	0%	0.48%	1	1	2	2	2	1	1	1	1	1	1	1	1	1	1	1	1	1	2	1	1	2	1	2	2	1	1	1	1	1	1	1	1	1	1	1	2	1	1	1	2	2	1	1	2	1	1	1	2	2	2
Haplo_59	0%	0%	0%	0%	0%	0%	0.48%	1	1	2	2	2	1	1	1	1	1	1	1	1	1	1	1	1	1	2	1	1	2	2	2	1	1	1	1	1	1	1	1	2	1	1	1	1	1	1	1	1	1	1	1	2	1	2	1	2	2	1
Haplo_60	0%	0%	0%	0%	0%	0%	0.48%	1	1	2	2	2	2	2	1	1	1	1	1	1	1	1	2	1	1	2	2	1	1	2	2	1	1	1	1	1	1	1	1	2	1	1	1	1	1	1	1	1	1	1	1	2	1	2	1	2	2	1
Haplo_61	0%	0%	0%	0%	0.50%	0%	0%	1	1	2	2	2	2	2	1	1	1	1	2	1	2	1	1	1	1	2	1	1	1	1	1	1	1	1	1	1	1	1	1	1	1	1	1	1	1	1	1	1	1	2	1	1	1	1	1	1	1	2
Haplo_62	0%	0.65%	0%	0%	0.74%	0%	0%	1	1	2	2	2	2	2	1	1	1	1	2	1	2	1	1	1	1	2	1	1	1	2	1	1	1	1	1	1	1	1	1	1	1	1	1	1	1	1	1	1	1	2	1	1	1	1	1	1	1	2
Haplo_63	0%	0%	0%	0%	0%	0%	0.95%	1	1	2	2	2	2	2	1	1	1	2	2	1	2	1	1	1	1	2	2	1	1	1	2	2	1	2	2	1	1	1	1	2	1	1	1	1	1	1	1	1	1	1	1	2	1	1	1	2	2	1
Haplo_64	0%	0%	0%	0%	0%	0%	0.48%	1	1	2	2	2	2	2	1	1	1	2	2	1	2	1	1	1	1	2	2	1	1	2	2	1	1	1	1	1	1	1	1	1	1	1	1	2	1	1	1	1	2	1	1	1	1	1	1	2	2	1
Haplo_65	0%	0%	0%	0.31%	0%	0%	0%	1	1	2	2	2	2	2	1	1	1	2	2	1	2	1	1	1	1	2	2	1	1	2	2	1	1	1	1	1	1	1	1	2	1	1	1	1	1	1	1	1	1	1	1	2	1	1	2	1	1	2
Haplo_68	0%	0%	0%	0%	0.50%	0%	0%	1	1	1	1	1	1	1	1	1	1	1	1	1	1	1	1	1	1	1	1	1	1	1	2	1	1	1	1	1	1	1	1	2	1	1	1	1	1	1	1	1	1	1	1	2	1	1	2	1	1	2

Haplotype nr	America	East Asia	Europe	Middle East	Central South Asia	Oceania	Subsaharian Africa	rs10265249	rs7458593	rs4723034	rs7384927	rs10225302	rs13438031	rs28371550	rs6954044	rs33995922	rs28371551	rs17159769	rs28371552	rs28371553	rs17159772	rs28371554	rs28371555	rs28371556	rs28371557	rs2302019	rs2302020	rs2302021	rs4988494	rs2267721	rs2267723
Haplo_69	0%	0%	0%	0%	0.50%	0%	0%	1	1	1	1	1	1	1	1	1	1	1	1	1	1	1	1	1	1	1	1	1	1	1	2
Haplo_7	0.78%	0%	0%	0%	0%	0%	0%	1	1	1	2	1	1	1	1	1	1	1	1	1	1	1	1	1	1	1	1	1	1	1	1
Haplo_70	0%	0%	0%	0.92%	0%	0%	0%	1	1	1	1	1	1	1	1	1	1	1	1	1	1	1	1	1	1	1	1	1	1	1	2
Haplo_71	0%	0%	0%	0%	0.25%	0%	0%	1	1	1	1	1	1	1	1	1	1	1	1	1	1	1	1	1	1	1	1	1	1	1	2
Haplo_73	0%	0%	0%	0.31%	0%	0%	0%	1	1	1	1	1	1	1	2	1	1	1	1	1	1	1	1	1	1	1	1	1	1	1	1
Haplo_74	0%	0%	0%	0.61%	0%	0%	0%	1	1	1	1	2	1	1	1	1	1	1	1	1	1	1	1	1	1	1	1	1	1	1	1
Haplo_75	0%	0%	0%	0%	0%	3.57%	0%	1	1	1	1	2	1	1	1	1	1	1	1	1	1	1	1	1	1	2	1	1	2	1	1
Haplo_76	0%	0%	0%	0.92%	0%	0%	0%	1	1	1	1	2	1	1	1	1	1	1	1	1	1	1	1	1	1	2	1	1	2	1	2
Haplo_77	0%	0%	0%	0%	0%	3.57%	0%	1	1	1	1	2	1	1	1	1	1	1	1	1	1	1	1	1	1	2	1	1	2	1	2
Haplo_78	0%	0%	0%	0.31%	0%	1.79%	0%	1	1	1	1	2	1	1	1	1	1	1	1	1	1	1	1	1	1	2	1	1	2	2	2
Haplo_79	0%	0%	0%	0%	1.49%	0%	0%	1	1	1	1	2	1	1	1	2	1	1	1	1	1	1	1	1	1	2	1	1	2	1	1
Haplo_8	5.47%	0%	0%	0%	0%	0%	0%	1	1	1	2	1	1	1	1	1	1	1	1	1	1	1	1	1	1	1	1	1	1	1	1
Haplo_80	0%	0%	0%	0%	0.25%	0%	0%	1	1	1	1	2	1	1	1	2	1	1	1	1	1	1	1	1	1	2	1	1	2	1	2
Haplo_81	0%	0%	0%	0%	1.24%	0%	0%	1	1	1	1	2	1	1	1	2	1	1	1	1	1	1	1	1	1	2	1	1	2	1	2
Haplo_82	0%	0%	0%	0%	0.25%	0%	0%	1	1	1	1	2	1	1	1	2	1	1	1	1	1	1	1	1	1	2	1	1	2	1	2
Haplo_84	0%	0%	0%	0%	0.25%	0%	0%	1	1	1	1	2	1	1	1	2	1	1	1	1	1	1	1	1	1	2	1	1	2	1	2
Haplo_85	0%	0.43%	0%	0%	0%	0%	0%	1	1	1	1	2	2	2	1	1	1	2	2	1	2	1	1	1	1	2	2	1	1	2	1
Haplo_86	0%	0%	0%	0%	0.25%	0%	0%	1	1	1	1	2	2	2	1	1	1	2	2	1	2	1	1	1	1	2	2	1	1	2	2
Haplo_87	0%	0%	0%	0.61%	0.25%	0%	0%	1	1	1	1	2	2	2	1	1	1	2	2	1	2	1	1	1	1	2	2	1	1	2	2
Haplo_89	0%	0%	0%	0%	0%	0%	0.48%	1	1	1	1	1	1	1	1	1	1	1	1	1	1	1	1	1	1	1	1	1	1	1	2
Haplo_90	0%	0%	0%	0%	0.25%	0%	0%	1	1	1	1	2	1	1	1	2	1	1	1	1	1	1	1	1	1	2	1	1	2	1	2
Haplo_91	0%	0%	0%	0%	0.50%	0%	0%	1	1	1	1	2	2	2	1	1	1	2	2	1	2	1	1	1	1	2	2	1	1	2	2
Haplo_93	0%	0%	0%	0%	0.25%	0%	0%	1	1	1	2	1	1	1	1	1	1	1	1	1	1	1	1	1	1	1	1	1	1	1	2
Haplo_94	0%	0%	0%	0%	0.50%	0%	0%	1	1	1	2	1	1	1	1	1	1	1	1	1	1	1	1	1	1	1	1	1	1	1	2
Haplo_95	0%	0.22%	0%	0%	0%	0%	0%	1	1	1	2	2	1	1	1	1	1	1	2	1	1	1	1	1	1	2	1	2	2	2	2
Haplo_96	0%	0%	0%	0.31%	0%	0%	0%	1	1	1	2	2	2	2	1	1	1	2	2	1	2	1	1	1	1	2	2	1	1	2	2
Haplo_99	0%	0.22%	0%	0%	0%	0%	0%	1	1	2	1	1	1	1	1	1	1	1	1	1	1	1	1	1	1	1	1	1	1	1	2

Haplotype nr	rs4988496	rs28371558	rs28371559	rs4988498	rs4988499	rs740336	rs28371560	rs2741	rs4988505	rs28371561	rs28371562	rs2228078	rs2586	rs28371563	rs28371564	rs28371565	rs28371566	rs6962369	rs10653361	rs4723039	rs28371567	rs28371568	rs28371569	rs28371570	rs11771444	rs11973385	rs11760522
Haplo_69	1	1	1	1	1	1	1	1	2	1	1	1	1	1	1	1	1	2	1	1	2	1	1	2	2	1	1
Haplo_7	1	1	1	1	1	1	1	1	1	1	1	1	1	1	1	1	1	1	2	1	1	1	1	2	1	1	2
Haplo_70	2	1	2	2	1	1	1	1	2	1	1	1	1	1	1	1	1	1	1	1	2	2	1	1	2	2	1
Haplo_71	2	1	2	2	1	1	1	1	2	1	1	1	1	2	1	1	1	1	1	1	2	1	1	1	1	1	2
Haplo_73	1	1	1	1	1	1	1	1	1	1	1	1	1	1	1	1	1	1	2	1	1	1	1	1	1	1	2
Haplo_74	1	1	1	1	1	1	1	1	1	1	1	1	1	1	1	1	1	1	2	1	1	1	1	1	1	1	2
Haplo_75	1	1	1	1	1	1	1	1	1	1	1	1	1	1	1	1	1	1	2	1	1	1	1	1	1	1	2
Haplo_76	1	1	1	1	1	1	1	1	2	1	1	1	1	1	1	1	1	1	2	1	1	1	1	2	1	1	2
Haplo_77	1	1	1	1	2	2	1	1	2	1	1	1	1	2	1	1	1	1	1	1	2	1	1	1	2	2	1
Haplo_78	1	1	1	1	2	2	1	1	2	1	1	1	1	2	1	1	1	1	1	1	2	1	1	1	2	2	1
Haplo_79	1	1	1	1	1	1	1	1	1	1	1	1	1	1	1	1	1	1	2	1	1	1	1	1	1	1	2
Haplo_8	1	1	1	1	1	1	1	1	1	1	1	2	1	1	1	1	1	1	2	2	1	1	1	1	1	1	2
Haplo_80	1	1	1	1	1	1	1	1	1	1	1	1	1	1	1	1	1	1	2	1	1	1	1	1	1	1	2
Haplo_81	1	1	1	1	1	1	1	1	1	1	1	1	2	1	2	1	1	2	1	1	2	1	1	1	2	2	1
Haplo_82	1	1	1	1	1	1	1	1	1	1	1	1	2	1	2	2	1	2	1	1	2	1	1	1	2	2	1
Haplo_84	1	1	1	1	2	2	1	1	2	1	1	1	1	2	1	1	1	1	1	1	2	1	1	1	2	2	1
Haplo_85	1	1	1	1	1	1	1	1	1	1	1	1	1	1	1	1	1	1	2	1	1	1	1	1	1	1	2
Haplo_86	1	1	1	1	1	1	1	1	2	1	1	1	1	1	1	1	1	1	1	1	2	1	1	2	1	1	2
Haplo_87	1	1	1	1	1	1	1	2	1	1	1	1	1	1	1	1	1	2	1	1	1	1	1	1	2	2	1
Haplo_89	1	1	1	1	1	1	1	1	2	1	1	1	1	1	1	1	1	1	1	1	2	1	1	2	1	1	2
Haplo_90	1	1	1	1	1	1	1	2	1	1	1	1	1	1	1	1	1	2	1	1	1	1	1	1	2	2	1
Haplo_91	1	1	1	1	1	1	1	2	1	1	1	1	1	1	1	1	1	2	1	1	1	1	1	1	2	2	1
Haplo_93	1	1	1	1	1	1	1	1	2	1	1	1	1	1	1	1	1	1	1	1	2	1	1	2	1	1	2
Haplo_94	1	1	1	1	1	1	1	1	2	1	1	1	1	1	1	1	1	2	1	1	2	1	1	1	1	1	1
Haplo_95	1	1	1	1	1	1	1	2	1	1	1	1	1	1	1	1	1	2	1	1	1	1	1	1	2	2	1
Haplo_96	1	1	1	1	1	1	1	2	1	1	1	1	1	1	1	1	1	2	1	1	1	1	1	1	2	2	1
Haplo_99	1	1	1	1	1	1	1	2	1	1	1	1	1	1	1	1	1	2	1	1	1	1	1	1	1	1	2
