# Supplementary material for: Extended Haplotypes in the Growth Hormone Releasing Hormone Receptor Gene (GHRHR) Are Associated with Normal Variation in Height
Source: PLoS One. 2009 Feb 11;4(2):e4464. doi: 10.1371/journal.pone.0004464 (PMC2637425; doi:10.1371/journal.pone.0004464)
Supplement: Table S3 — General description of the two population cohorts (0.05 MB DOC) [file pone.0004464.s003.doc]

| **Cohort** | **Females** | **Males** | **Total** | **No families** |  |  |  |
| --- | --- | --- | --- | --- | --- | --- | --- |
| VB | 267 | 244 | 511 | 119 |  |  |  |
| NB | 371 | 329 | 700 | 81 |  |  |  |
|  |  |  |  |  |  |  |  |
| **Height (cm)** | **Sex** | **Min** | **1st Qu.** | **Median** | **Mean** | **3rd Qu.** | **Max** |
| VB | Females | 141 | 155.5 | 161 | 160.7 | 165.5 | 178 |
| VB | Males | 150 | 170 | 175.5 | 174.9 | 181 | 200 |
| NB | Females | 140 | 154 | 159 | 158.7 | 164 | 175 |
| NB | Males | 148 | 166 | 172 | 171.1 | 176 | 189 |
|  |  |  |  |  |  |  |  |
| **Age (Year)** | **Sex** | **Min** | **1st Qu.** | **Median** | **Mean** | **3rd Qu.** | **Max** |
| VB | Females | 30 | 40 | 40 | 45.49 | 50 | 75 |
| VB | Males | 30 | 40 | 40 | 43.5 | 50 | 80 |
| NB | Females | 14 | 28 | 45 | 46.19 | 64 | 94 |
| NB | Males | 15 | 29 | 48 | 47.73 | 65 | 87 |

Table S3. General description of the two population cohorts
